# Single cell characterization of B-lymphoid differentiation and leukemic cell states during chemotherapy in ETV6-RUNX1-positive pediatric leukemia identifies drug-targetable transcription factor activities

**DOI:** 10.1186/s13073-020-00799-2

**Published:** 2020-11-20

**Authors:** Juha Mehtonen, Susanna Teppo, Mari Lahnalampi, Aleksi Kokko, Riina Kaukonen, Laura Oksa, Maria Bouvy-Liivrand, Alena Malyukova, Artturi Mäkinen, Saara Laukkanen, Petri I. Mäkinen, Samuli Rounioja, Pekka Ruusuvuori, Olle Sangfelt, Riikka Lund, Tapio Lönnberg, Olli Lohi, Merja Heinäniemi

**Affiliations:** 1 Institute of Biomedicine, School of Medicine, University of Eastern Finland, Yliopistonranta 1, FI-70211 Kuopio, Finland; 2 BioMediTech, Faculty of Medicine and Health Technology, Tampere University, FI-33014 Tampere, Finland; 3 Turku Bioscience Centre, University of Turku and Åbo Akademi University, FI-20520 Turku, Finland; 4 Department of Cell and Molecular Biology, Karolinska Institutet, SE-171 77 Stockholm, Sweden; 5 A.I. Virtanen Institute for Molecular Sciences, University of Eastern Finland, Yliopistonranta 1, FI-70211 Kuopio, Finland; 6 Fimlab Laboratories, FI-33520 Tampere, Finland; 7 Tays Cancer Centre, Tampere University Hospital, Tampere, Finland

**Keywords:** Cell differentiation, Leukemia, Gene regulation, Single cell genomics

## Abstract

**Background:**

Tight regulatory loops orchestrate commitment to B cell fate within bone marrow. Genetic lesions in this gene regulatory network underlie the emergence of the most common childhood cancer, acute lymphoblastic leukemia (ALL). The initial genetic hits, including the common translocation that fuses *ETV6* and *RUNX1* genes, lead to arrested cell differentiation. Here, we aimed to characterize transcription factor activities along the B-lineage differentiation trajectory as a reference to characterize the aberrant cell states present in leukemic bone marrow, and to identify those transcription factors that maintain cancer-specific cell states for more precise therapeutic intervention.

**Methods:**

We compared normal B-lineage differentiation and in vivo leukemic cell states using single cell RNA-sequencing (scRNA-seq) and several complementary genomics profiles. Based on statistical tools for scRNA-seq, we benchmarked a workflow to resolve transcription factor activities and gene expression distribution changes in healthy bone marrow lymphoid cell states. We compared these to ALL bone marrow at diagnosis and in vivo during chemotherapy, focusing on leukemias carrying the *ETV6-RUNX1* fusion.

**Results:**

We show that lymphoid cell transcription factor activities uncovered from bone marrow scRNA-seq have high correspondence with independent ATAC- and ChIP-seq data. Using this comprehensive reference for regulatory factors coordinating B-lineage differentiation, our analysis of *ETV6-RUNX1*-positive ALL cases revealed elevated activity of multiple ETS-transcription factors in leukemic cells states, including the leukemia genome-wide association study hit ELK3. The accompanying gene expression changes associated with natural killer cell inactivation and depletion in the leukemic immune microenvironment. Moreover, our results suggest that the abundance of G1 cell cycle state at diagnosis and lack of differentiation-associated regulatory network changes during induction chemotherapy represent features of chemoresistance. To target the leukemic regulatory program and thereby overcome treatment resistance, we show that inhibition of ETS-transcription factors reduced cell viability and resolved pathways contributing to this using scRNA-seq.

**Conclusions:**

Our data provide a detailed picture of the transcription factor activities characterizing both normal B-lineage differentiation and those acquired in leukemic bone marrow and provide a rational basis for new treatment strategies targeting the immune microenvironment and the active regulatory network in leukemia.

## Background

Failures in lymphoid cell differentiation underlie the emergence of acute lymphoblastic leukemia (ALL) that peaks in incidence in childhood [1]. Recently, 35 potential cell states in hematopoiesis were resolved using single cell RNA-seq (scRNA-seq) data based on eight healthy bone marrow (BM) donors profiled by the Human Cell Atlas (HCA) groups, comprising approximately 100,000 cells [2]. Understanding normal B cell differentiation in BM forms the basis to characterize the aberrant cell states in cancers that originate from lymphoid progenitor cells. Previous work has identified tight regulatory loops that orchestrate B cell fate [3]. However, their activity along the single cell resolution trajectory in human B-lineage has not been studied in detail.

The genetic basis of ALL initiation and progression is mechanistically linked to alterations in key lymphoid transcription factors (TFs) [1]. The most common translocation t(12;21) generates a fusion between two TFs: the repressive domain of ETV6 is fused with RUNX1, retaining the RUNT-DNA-binding domain. This confers cells with functional properties that sustain self-renewal and survival [4]. We and others have shown that the aberrant ETV6-RUNX1 (E/R) TF fusion can silence key genes and regulatory regions [5–9]. In effect, cells become arrested at a lymphoid progenitor state [7, 10], whereby additional DNA lesions can accumulate, which especially in E/R leukemias are driven by a transcription-coupled mechanism that results in off-targeting of the recombination activating gene (RAG) complex [11, 12]. However, the emerging cell state heterogeneity that manifests at diagnosis and during chemotherapy within the bone marrow remains poorly characterized.

In the clinics, the accumulated knowledge regarding initiating genetic lesions has been implemented into diagnostic screens that inform choices between chemotherapy regimes that differ in intensity. However, almost half of relapses occur in children presenting initially with good-risk cytogenetic features such as E/R [13], thus raising the question what underlies their resistance. Epigenetic changes driven by TF, coregulator, and chromatin modifier activities in the blast cells contribute to the blast cell phenotype [14, 15]. The epigenetic plasticity of leukemic cells may support resistant states [16, 17] and allow conversion into quiescent stem-like states or lineage switching to escape the cytotoxic agents [18–22]. This poses a challenge in the design of drug therapy and urges the development of new therapies informed by characterization of the cancer cells and their cross-talk with the microenvironment.

Single cell genomics holds promise to resolve the leukemic gene regulatory programs even in small cell populations, based on mRNA, chromatin, and DNA profiles [23]. Computational analysis can resolve TF activity and transcriptome dynamics and capture changes in gene expression distributions between cell states analyzed [24–26]. Here, we set out to elucidate cell states and TF activities characteristic of normal B-lineage differentiation from hematopoietic stem cells (HSCs) and to compare these to the *E/R+* ALL cases at diagnosis and during standard chemotherapy.

## Methods

### Patient samples

This study was approved by the Regional Ethics Committee in Pirkanmaa, Tampere, Finland (#R13109), and conducted according to the guidelines of the Declaration of Helsinki. A written informed consent was received by the patient and/or guardians. All the patients were positive for the E/R-fusion transcript based on clinical RT-qPCR and FISH analysis (further confirmed using bulk WGS data). Their age ranged between 1 and 10 years, and all cases received standard induction therapy according to the NOPHO ALL-2008 protocol, with prednisolone 60 mg/m^2^/day p.o. days 1–28; vincristine 2.0 mg/m^2^ i.v. days 1, 8, 15, 22, and 29; doxorubicin 40 mg/m^2^ i.v. days 1 and 22; and methotrexate i.t. days 1, 8, 15, and 29 [27]. Leukemic blast percentages in the bone marrow during diagnosis, at day 15, and at day 29 are shown in Table 1. All the samples were CD19+, CD22+, CD10+, TdT+, cyCD79a+, and CD34+ (ALL9 and ALL3 heterogenously), as measured by flow cytometry at diagnosis (Additional file 1, Fig. S4e). Mononuclear cells (MNCs) were extracted from fresh bone marrow (BM) using Ficoll-Paque Plus (GE Healthcare, #17-1440-02). Bone marrow MNCs were also extracted from two patients (ALL10 and ALL12) during the induction therapy at day 15 after initiation of therapy. MNCs were viably frozen in 15% DMSO/40% FBS in RPMI in liquid nitrogen. In addition, nuclei from samples ALL7 and ALL13 were isolated for global run-on sequencing (GRO-seq) as described in [5], snap-frozen, and stored at − 80 °C in a freezing buffer containing 40% glycerol.

**Table 1:** Leukemic blast percentages in clinical bone marrow samples of the E/R-positive patients during induction therapy determined by flow cytometry

Sample ID	Leukemic blast percentage at diagnosis	Leukemic blast percentage at day 15	Leukemic blast percentage at day 29
ALL1	94	10	0.3
ALL3	95	74	0.16
ALL8	93	0.93	0.02
ALL9	79	0.17	0.01
ALL10	65	10	0.08
ALL12	90	59	0.2
ALL7	27	0	0
ALL13	80	0.04	0

### Cell line samples

The *E/R+* REH cell line (ACC-22, DSMZ, Germany) was maintained in RPMI 1640 (Gibco, Thermo Fisher) supplemented with 10% FBS (Gibco, Thermo Fisher), 2 mM l-glutamine (Gibco, Thermo Fisher), penicillin (100 U/ml), and streptomycin (100 mg/ml) (Sigma-Aldrich). Mycoplasma status was defined negative for all cell lines by PCR (PCR Mycoplasma Test Kit I/C, PromoCell GmbH, Germany), and cell lines were authenticated by Short Tandem Repeat genotyping (Eurofins Genomics, Ebersberg, Germany).

### scRNA-seq

Single cell gene expression was studied to characterize leukemic bone marrow cell populations (for datasets analyzed, see Additional file 2, Table S1). Cells from primary BM samples (*n* = 6 diagnostic, *n* = 2 post-treatment) were processed for scRNA-seq in the Finnish Functional Genomics Center, Turku, Finland, in 4 batches: (1) ALL3, (2) ALL1, (3) ALL10 and ALL10-d15, and (4) ALL8, ALL9, ALL12, and ALL12-d15. Before applying the cells into the Chromium cartridge, their viability was checked using Trypan blue. PI-negative (live) cells were selected from sample ALL3 using FACS. Samples ALL1, ALL10, and ALL10-d15 were processed directly after thawing the MNC fraction without further processing. The diagnostic samples from these were also analyzed using flow cytometry to compare the detected leukemic cell fraction in the thawn ampoules and in the final scRNA-seq data matrix (see Additional file 2, Table S1). The CD19+ cell percentages in samples that were FACS-sorted (ALL03 batch 1), processed directly after thaw (ALL01 batch 2), or processed with dead cell removal kit (ALL10 batch 3) were highly concordant between scRNA-seq and Amnis flow cytometry. Excess dead cells were depleted from samples ALL8 and ALL9 using bead-based Dead Cell Removal Kit (#130-090-101, MACS Miltenyi Biotech), increasing the percentage of viable cells from 43 to 72% and from 63 to 78%, respectively. For samples ALL12 and ALL12-d15, enrichment of leukemic cells was carried out by depleting non-B cells using streptavidin beads (BD Streptavidin Particles Plus, BD Biosciences, Franklin Lakes, NJ, USA) and biotinylated antibodies against human CD16 (clone 3G8), CD14 (HCD-14), CD11c (3.9), CD56 (HCD56), CD3 (UCHT1), and CD66 (G10F5) (Biolegend), all with final concentrations of 2 μg/ml, following the manufacturer’s instructions. Depletion efficiency was estimated by flow cytometry using CD3 (BV421, BD Biosciences, #56287, RRID:AB_27378607) and CD19 (Thermo Fisher Scientific, #25-0199-41, RRID:AB_1582279) antibodies, with a viability dye (eBioscience, Fixable Viability Dye eFluor™ 506, #65-0866-14). Depletion decreased the proportion of T cells (CD3+) from 30 to 2%, increased the proportion of B cells (CD19+) from 23 to 50%, and increased the percentage of viable cells from 50 to 80% in a test BM sample.

scRNA-seq was performed using the 10X Genomics Chromium technology, according to the Chromium Single-Cell 3′ Reagent Kits V2 User guide Rev B. In brief, cells were combined with reverse transcriptase Master Mix and partitioned into Gel Bead-In EMulsions (GEMs) using 10X GemCode Technology, where the poly-A transcripts are barcoded with an Illumina R1 sequence, a 16-bp 10X barcode and a 10-bp Unique Molecular Identifier (UMI). Eleven to 12 cycles of PCR was used to amplify the cDNA. Sequencing was performed using the Illumina HiSeq 3000. Primary BM samples were sequenced to an average depth of ~ 50,000 reads per cell.

For the analysis of drug treatment at single cell level, REH cells were seeded into 6-well plates (0.6 million/ml concentration) and treated with XRP44X (Sigma-Aldrich) (1 μM), TK216 (MedChemExpress, NJ USA) (800 nM), or DMSO for 72 h. After treatment, cells were collected and their viability was checked using Trypan blue with Cellometer Mini Automated Cell Counter (Nexcelom Bioscience) and Dead Cell Removal Kit (#130-090-101, MACS miltenyi Biotech) was used per the manufacturer’s protocol. Viable cells were eluted by rinsing twice with 1 ml binding buffer. Cell viabilities were increased from DMSO 95%, XRP44X 79%, and TK216 76% to 97%, 94%, and 96%, respectively. Subsequently, 0.42–0.5 million cells were methanol fixated according to 10X Genomics Methanol Fixation of Cells for Single Cell RNA Sequencing protocol User guide CG000136 Rev E, using a mix of two RNAse inhibitors (RNase Inhibitor, Thermo Fisher, Carlsbad, CA, USA, and RNasin® Plus RNase Inhibitor, Promega, Madison, WI, USA) and DTT (Thermo Fisher, Carlsbad, CA, USA). scRNA-seq was performed using the 10X Genomics Chromium technology, according to the Chromium Single Cell 3′Reagent Kits v3 User guide CG000183 Rev C with loading concentration of 2100–2200 cells/μl. Sequencing was performed in Novogene (UK) Company Limited, Cambridge, UK, with a PE150 NovaSeq sequencer, aiming at 50,000 reads per cell.

### HCA bone marrow scRNA-seq data processing and cell state annotation

Characterization of normal bone marrow B-lymphoid cell states was performed using data from healthy donors (*n* = 8), available from the HCA data portal. Raw fastq files corresponding to 10X Genomics Chromium single cell data were downloaded from [28]. Data was aligned with Cell Ranger 3.0.2 to human reference (hg19) version 3.0.0 with default parameters, and the filtered count matrix was taken for downstream analysis (for Cell Ranger quality control summaries, refer to Additional file 2, Table S1). Scanpy [29] (version 1.4) was used for initial characterization of cells [30] as follows: Genes were first filtered to include only genes present in more than 100 cells. Then, bad quality cells were removed if (i) UMIs arising from mitochondrial genes in a cell accounted for more than 10% of total UMI count, while possible doublets were excluded based on (ii) total number of UMIs 50,000 or more, or (iii) the number of genes expressed in a cell 6000 or more. Next, genes were filtered once more to include only those expressed in minimum 400 cells. UMI count data was then normalized to relative counts per cell by dividing by the total count per cell and then scaling by a factor of 10,000. Highly variable genes (HVGs) were defined as genes with minimum mean expression 0.0125, maximum mean expression 3, and minimum dispersion 0.5, resulting in 2046 genes with the rest of the genes filtered out from the data for downstream analyses. To reduce undesired technical effects in data analysis, we regressed out the effect of the number of UMIs and the percentage of UMIs arising from mitochondrial genes to gene expression in each cell. Mutual nearest neighbor (MNN) correction [31] (mnnpy [32] version 0.1.9.5) was used to combine data across the eight donors for clustering and cell state identification. Principal component analysis (PCA) was then calculated using the processed data (Scanpy version 1.4). Top 50 principal components (PCs) were used to calculate a neighborhood graph (the number of neighbors was set to 30) that was used as input for Uniform Manifold Approximation and Projection (UMAP) [33], where the effective minimum distance between embedded points was set to 0.5, and Louvain clustering [34] with resolution set to 1.0, which was enough to characterize major cell type clusters from the data. Wilcoxon’s test was used to find marker genes for each cluster which were used to characterize the found clusters in concordance with known marker genes. Cell cycle states (G1, S, G2/M) of cells were annotated by scoring gene sets with Scanpy using annotated cell cycle genes from [35].

To focus on B-lineage cell differentiation, a subset of cells from clusters containing hematopoietic stem cells and B cell lineage cells was re-analyzed in an iterative manner, each time running the basic workflow again with additional filtering steps. Initially, genes expressed in less than 100 cells were removed when analyzing this subset. When choosing highly variable genes, we required the minimum dispersion to be 1, compared to the previous 0.5 to obtain a smaller set of HVGs in attempt to identify potential outliers. Small clusters containing high expression of markers for T cells, NK T (natural killer T) cells, monocytes, and erythroid precursor cells were still present after the first iteration and were filtered out. In the second iteration, we required the minimum mean expression to be 0.1 and the minimum dispersion 0.5 for choosing highly variable genes. In the neighborhood graph calculation, the number of principal components used was here set to 20 as we presumed the lower number of PCs is sufficient to capture the variance between these cell types. Next, we filtered each cluster for possible outliers by calculating cluster-specific Median Absolute Deviance (MAD) for number of UMIs and percentage of UMIs from mitochondrial genes and removed cells assigned to the cluster with MAD greater than 5 in either. This was motivated by the large differences between clusters in these metrics. During B cell differentiation, the cells display marked changes in cell size (e.g., transitioning from large cycling pre-B cells to small pre-B cells). Thus, this choice is also motivated by biology. With the filtered subset of 20,753 cells, we ran through the workflow once again, choosing highly variable genes with minimum mean 0.1 and minimum dispersion 0.75 and setting the number of principal components in neighborhood graph calculation to 20. The final clusters were characterized as described above.

### ALL scRNA-seq data processing and cell state annotation

To perform similar analysis in leukemic BM, raw patient data acquired in this study (*n* = 6 diagnostic, *n* = 2 post-treatment) was processed and aligned with Cell Ranger (version 3.0.2) with the same settings as the HCA data (for Cell Ranger quality control summaries, refer to Additional file 2, Table S1). Scanpy (version 1.4) was used for initial characterization of cells following the same approach as outlined above [30] (HCA analysis): Genes were first filtered to include only genes present in more than 100 cells, requiring this metric to exceed 200 in the final iteration. Cells were removed if (i) UMIs arising from mitochondrial genes in a cell were more than 10%, (ii) the total number of UMIs was 40,000 or more, or (iii) the number of genes expressed in a cell was 5000 or more. UMI count data was then normalized to relative counts per cell by dividing by the total count per cell and then scaling by a factor of 10,000. Highly variable genes were defined as genes with minimum mean expression 0.0125, maximum mean expression 3, and minimum dispersion 0.5, resulting in 1425 genes that were used for clustering and dimensionality reduction (50 principal components, number of neighbors 15, resolution 1.0). The number of neighbors was set lower than with HCA data as the total number of cells is lower in these data. MAD filtering was used to remove outlier cells from clusters, as described above. With the final cell subset passing these criteria (44,746 cells), the workflow was repeated and clusters characterized based on marker genes.

### Pediatric BM scRNA-seq data processing

Single cell RNA-seq data from three pediatric BM donors [36] was downloaded from NCBI GEO [37] and processed similarly as the HCA BM data to enable independent validation of results. The data was aligned with Cell Ranger 3.0.2 to human reference (hg19) version 3.0.0 and the resulting count matrix was subject to processing with Scanpy [29] following the exact same workflow as HCA. In short, low quality cells (more than 10% of UMIs from mitochondrial genes, more than 30,000 UMIs, or more than 4000 genes expressed) were filtered out and genes expressed in less than 40 cells were removed before normalizing cells by dividing them with the total number of UMIs and then scaling with a factor of 10,000. Then, log-transformed normalized counts were used to find highly variable genes with mean expression between 0.0125 and 3 with minimum dispersion of 0.5, resulting in 2531 HVGs. The effect of number of UMIs and percentage of UMIs from mitochondrial genes per cell was regressed out before mutual nearest neighbor (MNN) batch correction where we set the three different donors as separate batches. After, PCA was calculated and a neighborhood graph was calculated by setting the number of PCs to 50 and number of neighbors to 30. Louvain clustering was run with resolution set to 1 using the neighborhood graph. Label transfer was run, using the cell types defined in HCA BM, and clusters corresponding to HSCs and B-lineage cells were extracted for a second iteration of the workflow. Starting from raw data, the counts were normalized and HVGs were defined as genes with mean normalized expression between 0.0125 and 3 and minimum dispersion of 0.75 resulting in 1929 HVGs. The effect of the number of UMIs and the percentage of UMIs from mitochondrial genes per cell was again regressed out before MNN batch correction. The neighborhood graph was calculated by setting the number of neighbors to 15 and the number of PCs to 20, to account for the smaller subset of the data. Louvain clustering with resolution set to 1 was calculated using the neighborhood graph. MAD filtering was applied for each cluster of cells, filtering out cells with MAD difference in number of UMIs per cell or percentage of UMIs from mitochondrial genes greater than 5. The remaining cells were used to perform downstream analyses.

### Healthy BM CITE-seq data processing

CITE-seq data from an independent adult healthy BM sample [38] were downloaded from NCBI GEO [39] and processed with Scanpy (similar settings as for HCA BM initial processing) for label transfer and UMAP visualization of B-lineage cell states.

### REH cell line drug treatment scRNA-seq

The REH cell line scRNA-seq libraries with drug treatments were aligned with Cell Ranger 3.1.0 using a combined human (hg19) and mouse (mm10) genome as reference. The human cells, corresponding to REH cells, were extracted from filtered count matrices using the Cell Ranger classification result. Cells that had (i) more than 2000 and less than 6000 UMIs, (ii) less than 20% of total UMIs arising from mitochondrial genes, and (iii) less than 500 UMIs aligned to mouse genes were kept in the analysis. Then, the data was log-normalized using a scaling factor of 10,000 and the normalized data was scored for cell cycle phases using functions in Seurat [40] (version 3.1.1).

### Differential distribution of read counts: scDD analysis

The gene expression distributions in subsequent cell states representing B-lineage differentiation, or between leukemic and normal cell states, were analyzed with the scDD package [26, 30]. As an independent validatory analysis, the matching cell clusters from the pediatric BM were identified using label transfer (Seurat, see below) and the same analyses repeated. The tool enables comparisons based on differential distribution and proportion of zeros between two groups of cells. Genes were assigned into three main categories—DE, DM, and DZ. DM and DE characterize changes in the expression distribution in cells with non-zero count for the gene analyzed (differential mean and differential modality, respectively). DZ genes differ between the groups in proportion of cells with zero read count for the gene analyzed. In the context of differentiation, where cells switch genes on/off to proceed in maturation, this metric was estimated to capture the most relevant changes.

To account for differences in the number of UMIs and genes detected in different cell types, variance stabilizing transformation [41] (version 0.2.0) was used to correct for these differences before differential distribution testing. Sample was used as the batch interaction term, and logarithm of UMI counts per cell was specified as the latent variable to regress out. The resulting corrected UMI counts were then used as input to scDD. When running scDD, we noticed that for some genes, the clustering of the expression level within scDD failed due to zero variance. To overcome this, the scDD tool was modified to add a small random number (sampled from a uniform distribution ranging from − 0.01 to 0.01) to counts for genes which had this problem [42]. Cells with 3000–3500 counts after the corrections were included in comparing the pre-B G1 vs. pro-B G1, and the pro-B G1 vs. leukemic G1 cells. The following numbers of cells per differentiation/disease state were compared: HSC_HCA_, 3660; early B-lymphoid_HCA_, 895; pro-B cycling_HCA_, 794; pro-B G1_HCA_, 1413; pre-B cycling_HCA_, 1714; pre-B I G1_HCA_, 2541; pre-B II G1_HCA_, 2025; diagnostic leukemic G1, 6340; diagnostic leukemic cycling, 7054; HSC_Caron_, 192; early B-lymphoid_Caron_, 60; pro-B cycling_Caron_, 98; pro-B G1_Caron_, 224; pre-B cycling_Caron_, 471; pre-B I G1_Caron_, 725; and pre-B II G1_Caron_, 351.

Further filtering for scDD results was done using adjusted *p* value and fold change or difference in percentage cutoffs (see Additional file 1: Fig. S2a-d). *p* values were adjusted using the Benjamini-Hochberg FDR method.

### Clustering genes based on differential zero proportion

Differentially distributed genes from the leukemic vs. pro-B zero proportion comparisons, present in both G1 and cycling cell-based comparisons (90 downregulated and 272 upregulated), were clustered based on their zero proportion metric in ten cell states (HSC, early lymphoid progenitors, pro-B cycling (S/G2/M), pro-B G1, pre-B cycling, pre-B G1 I, pre-B G1 II, immature B, leukemic cells G1, and leukemic cell cycling). *K*-mean centroids were calculated using the R package flexclust [43] (version 1.4-0) with *k* = 8 and correlation as distance metric using the kccaFamily function. Initially different numbers of clusters were tested (*k* = 6 to 10) to select *k* that resulted in distinct cluster centroid profiles with well-matching profiles for assigned genes.

### Pathway enrichment analysis

Gene lists were analyzed for enrichment of ontology and pathway terms using the online web server Enrichr [44, 45] (release January 2019). The analysis was performed based on gene sets from GO, MGI Mammalian Phenotype, Bioplanet, Reactome, and transcription factor (TF) perturbations. The tool provides several significance metrics. The combined score used for ranking here refers to the combination of *p* value (Fisher’s exact test) and the *z*-score that represents the deviation from the expected rank. Enriched terms were selected based on the combined score (> 150) cutoff. TF motif enrichment results from Genome Browser PWMs were selected based on nominal *p* value < 0.05 due to overall lower scores across gene lists analyzed.

### Ordering cells based on pseudotime

Pseudotime analysis can be used to find a latent trajectory (pseudotemporal ordering of cells) in single cell data, corresponding to differentiation or cell cycle. HSC and B-lineage cells from HCA BM data were subjected to pseudotime analysis following the best practices workflow by Luecken and Theis [46] using Scanpy (version 1.4.5). Non-expressed genes (zero UMIs in any cell) were excluded, and the data was normalized with size factors calculated using the scran package [47, 48] (version 1.10.2) where Louvain clusters (resolution 0.5) were used. The analysis was done two ways: using highly variable genes or selecting differentially distributed genes from our scDD analyses between HSC and B-lineage cell types and the cell cycle phase marker genes. Neighborhood graph was calculated with the number of principal components set to 15 and the number of neighbors set to 15. Diffusion map representation [49] was then calculated obtaining 15 diffusion components, and a pseudotime ordering was calculated using diffusion pseudotime [50] using 10 diffusion components and setting the required root cell as the HSC with the highest value in the 1st diffusion component (DC1). For visualization, the DC1 vector was mirrored to obtain a left to right pseudotime trajectory of cells. The ordering of clusters was highly comparable with HVG or custom gene selection. The latter is shown in figures for consistency.

### RNA dynamics analysis

During differentiation, dynamic changes occur in gene transcription that can be modeled based on newly synthesized RNA (reads corresponding to unspliced mRNA) and processed RNA (reads corresponding to mRNA). Based on the dynamic RNA processing model, predictions of the future transcriptome state can be obtained and visualized together with the measured current state. Velocyto CLI [25] (version 0.17.17) was used to calculate spliced and unspliced counts per gene using human reference genome (hg19) version 3.0.0 for Cell Ranger from 10X Genomics. Expressed repetitive elements were masked using expressed repeat annotation for hg19 downloaded from UCSC Genome Browser [51]. scVelo package [52] (version 0.1.21) was used to analyze RNA dynamics in B cell differentiation. The gene expression matrix was accompanied with the spliced and unspliced count matrices of HSCs and B-lineage cells from HCA BM data. The data was first filtered by removing genes with less than 10 shared UMI counts in both spliced and unspliced data. The matrices were each then normalized by dividing the counts in each cell with the median of the total counts per cell. The 3000 most variable genes were extracted based on the spliced count matrix and the data matrices were log-transformed. Thirty top PCs were defined based on the most variable gene spliced count data followed by neighborhood graph calculation, with the number of neighbors set to 30. Based on the neighborhood connectivities, the first order moments for spliced and unspliced matrices were calculated. The normalized unspliced and spliced count matrices were then used to estimate the velocity of each cell using the deterministic model. The velocities were embedded on a UMAP embedding which was calculated with the same pre-processing steps before calculating the diffusion map.

### Regulon discovery and transcription factor activity scoring

For the discovery of TF activities that characterize specific cell states, a modified SCENIC workflow [24, 30] was developed based on the python implementation of the SCENIC method [53]. In our implementation, equal amounts of cells per cell type were sampled from the original data to ascertain that differences in cell type abundances do not bias the analysis. Secondly, a small number randomly sampled from a uniform distribution ranging from − 0.01 to 0.01 was added to zero counts to help SCENIC identify repressive TF targets with higher precision as the original workflow identified the targets based on Pearson’s correlation of only cells with non-zero counts. This could reduce the number of cells used in the correlation calculation in worst cases close to zero, making the results unrobust. Thirdly, the discovered regulons were evaluated based on a left-out test set. Specifically, the input matrix (equal representation of cell types) was split into training (70% of cells) and test (30% of cells) sets. The default SCENIC pipeline for regulon discovery was then run for the training set. The regulons found were scored in the training and test sets, and the average score per cell type calculated in both sets. These mean regulon scores across cell types were compared between training and test sets with Pearson’s product moment correlation coefficient. Regulons with *p* value > 0.001 were discarded. The discovery was repeated 10 times. The final set of regulons was then scored using the whole original dataset. Because different iterations often find regulons with the same driving TF and a similar target gene set, the mean score of the regulon for each cell was used in downstream analysis. In these analyses, leukemic cells from different donors and collection times were treated as separate cell types. For filtering regulons, a linear model was fit 100 times per regulon to a subset of the regulon score matrix where 600 cells per cell type were sampled randomly from the original dataset. In the model, the response is the regulon score and the cell type label is the independent variable (score ~ cell type). Regulons with the mean coefficient of determination (*R*^2^) < 0.5 were considered to not show sufficient variation between cell types and were therefore filtered out. Additionally, a regulon was filtered out if the mean score in any cell type was above 70% percentile while the TF gene expression had > 96% of zeros, indicating not enough evidence for high regulon activity. Additionally, regulons with Pearson’s correlation less than − 0.8 to the TF gene expression were filtered out.

### Cell type assignment of ALL cells with label transfer

Annotated HCA BM cells were used as a reference to label the other non-leukemic and ALL scRNA-seq data. This was performed with label transfer functions from Seurat [40] (version 3.1.4) as follows: Each ALL sample was separately normalized with CPM with scale factor of 10,000 and then log-transformed followed by extracting top 2000 most variable genes. Then, separately for each ALL sample, transfer anchors between reference and sample were calculated, where the first 30 dimensions of CCA were used as neighbor search space. Finally, the leukemic cells were annotated with 30 first PCs used in the weighting procedure.

### Natural killer (NK) cell scRNA-seq analysis

Clusters labeled NK and NK T cells from full HCA BM and primary ALL data were combined and processed together starting from raw counts with Scanpy (version 1.4.5). Genes were first filtered to include only genes present in more than 100 cells. Then, cells were removed if (i) UMIs arising from mitochondrial genes in a cell were more than 5%, (ii) the total number of UMIs was below 500 or 3000 or more, or (iii) the number of genes expressed in a cell was below 200 or 3000 or more. Then, data was normalized with following the same steps and parameters as in the pseudotime analysis followed by extraction of 3000 most variable genes which were used to calculate the first 50 PCs followed by neighborhood graph calculation with the 50 PCs and number of neighbors set to 15. Leiden clustering [54] with resolution 1 was calculated identifying two clusters with high expression of erythroid markers *HBA1*, *HBA2*, and *HBB* which were then removed and analysis repeated starting from calculating the most variable genes. UMAP embedding was calculated with the obtained PCs and the neighborhood graph to visualize the data. Leiden clustering was calculated again but with resolution parameter set to 2 to obtain more detailed clusters. NK clusters were identified as *GNLY*+ or *NKG7*+ clusters (additional CD3 positivity distinguishing NK T cells). The cell barcodes from the NK clusters were used to calculate the percentage of NK cell from total MNC, or non-leukemic MNC. Marker genes for NK clusters were calculated with the Wilcoxon's test and discarding genes with fold change less than 2. Top 5 genes per cluster based on test score were extracted. Scores for NK subtype gene sets from [55] were calculated using the top 20 genes per gene set sorted by log-fold change (omitting NK T clusters).

### Bulk pro-B cell ATAC-seq analysis

For analyzing open chromatin regions in pro-B cells, ATAC-sequencing (assay for transposase-accessible chromatin) data of human fetal pro-B cells (*n* = 3) were retrieved from NCBI SRA database, GSE122989 [56]. Data pre-processing and peak calling were done following the ENCODE pipeline for ATAC-seq [57] (version 1.5.4) which is a tool for statistical signal processing and produces alignment and measures of enrichment. Caper configuration file was set up for the local server platform, and parameters in the JSON file were selected based on the example JSON file. Hg19 was used as a reference genome in alignment. Narrow peaks were pooled and merged from three replicates. The highest enriched 10,000 peaks were taken to downstream analysis. Regions overlapping annotated transcription start sites (TSS) (NCBI RefSeq and UCSC Known gene) were discarded. TF motif discovery was performed with HOMER [58] (version 4.9.1) findMotifsGenome.pl (-size 200 -mask) using the remaining (3923) open chromatin regions. *p* values were adjusted using the Benjamini-Hochberg FDR method.

### GRO-seq assay

To study enhancer and gene region activity, primary ALL BM samples (*n* = 2) were collected for global run-on sequencing (GRO-seq). In addition, our existing data in REH cells available via NCBI GEO (GSE67540 [59]) were analyzed. For these samples and ALL7, the nuclear isolation and library preparation protocols were performed as described in [12]. Briefly, run-on products labeled with BrUTP were extracted with TRIzure (Bioline, London, UK). RNA was precipitated first for 30 min at room temperature and then for extra 10 min on ice. Poly-A tailing reaction was carried out and nascent RNA collected using anti-BrUTP beads. The anti-BrUTP beads used previously [12] were not available for the collection of run-on products for ALL13, and for this sample, the libraries were performed as described in [60] with few modifications. Bead binding was performed using 30 μl of Protein G Dynabeads (Thermo Fisher Scientific Baltics UAB, V.A. Graiciuno 8, LT-02241 Vilnius, Lithuania) per sample with 2 μg anti-BrdU monoclonal antibody (cat# ab6326, Abcam, Cambridge, UK). Beads were washed four times with 300 μl of PBST wash buffer including SUPERase In RNase Inhibitor (Thermo Fisher, Carlsbad, CA, USA). The purified run-on RNAs were next converted to cDNA and PCR amplified for 13 cycles and selected to 225–350 bp length. Single-end sequencing (50 bp) was performed with Illumina Hi-Seq2000 (GeneCore, EMBL Heidelberg, Germany).

### GRO- and ChIP-seq data pre-processing

TF ChIP-seq was used to validate TF-target associations obtained using SCENIC. ChIP-seq data representing PAX5 and EBF1 (GSE126300 [61]) were available in hg19, while BCL11A (GSE99019 [62]) read data was processed to hg19 from raw reads. For BCL11A and GRO-seq data, the raw sequencing reads were quality controlled using the FastQC tool [63]. Bases with poor quality scores were trimmed (min 97% of positions have a min phred quality score of 10) using the FastX toolkit [64]. Duplicate reads were collapsed from ChIP-seq files using fastx (collapse), while reads mapping to rRNA regions (AbundantSequences as annotated by iGenomes) were discarded from GRO-seq data. The Bowtie software [65] (version 0.12.9 for GRO-seq, version 1.2.3 for ChIP-seq) was then used for alignment of remaining reads to the hg19 genome version, allowing up to two mismatches and no more than three matching locations. The best alignment was reported. Reads overlapping with the so-called blacklisted regions that include unusual low or high mappability as defined by ENCODE, ribosomal and small nucleolar RNA (snoRNA) loci from ENCODE, and a custom collection of unusually high signal depth regions from GRO-seq were used to filter the data. Subsequently, data was analyzed using HOMER [58] (version 4.9.1). GRO-seq tagDirectories were generated with fragment length set to 75 and data visualized using makeMultiWigHub.pl with strand specificity. HOMER [58] (version 4.9.1) findPeaks tool (-style factor) was used in peak calling from ChIP-seq against input sample.

### ChIP-seq peak analysis

The peak data was ranked based on peak calling statistics (lowest rand corresponding to best peak) and the rank annotated in each peak name. Next, peaks were associated with nearby genes using the approach described in [66]. The data was summarized by gene, recording the number of associated peaks, the peak ranks, and the peak distances to gene TSS.

### Bulk RNA-seq

RNA was extracted from diagnostic BM samples collected in PAXgene blood RNA tubes using PAXgene Blood RNA kit (cat #762174, Qiagen GmbH, Hilden, Germany), following the version 2 instructions for manual purification. In order to have high detection of both coding and non-coding transcripts, samples were processed with Globin-Zero Gold rRNA Removal Kit (Illumina) and directional libraries were prepared using NEBNext Ultra Directional RNA Library Prep kit (New England Biolabs). The library preparation and paired-end (150 bp) sequencing were performed by Novogene (HK) Company Limited (Hong Kong, China) using Illumina Novaseq 6000 aiming at 70 million read pairs per sample. Sequencing quality was controlled using the FastQC tool, and reads were aligned to hg19 using STAR 2.5.1b, providing an annotated genome reference (Gencode v32lift37). The splice junctions discovered in each run were combined across samples analyzed and used to update the genome reference for 2-pass alignment. The aligned reads were visualized using IGV as coverage tracks and Sashimi plots.

To compare *E/R*+ cases to other ALL subtypes based on bulk RNA-seq data, the Pan-ALL dataset [67] consisting of 1988 samples representing various ALL subtypes was downloaded as regularized log-transformed values. A two-sided Wilcoxon rank sum test was calculated between the E/R subtype and the rest of the samples on selected genes, and the *p* values were corrected with the Benjamini-Hochberg FDR method.

### Immunofluorescence stainings and flow cytometry

For studying cell surface CD19 and RNA probe intensities in leukemic bone marrows, 0.2–0.5 million viably frozen mononuclear cells were first blocked using FcR Blocking Reagent (Miltenyi Biotech, #130-059-901, lot 5170502354) for 5 min. Staining with anti-CD19 (PECy7-conjugated, Invitrogen, Thermo Fisher, # 25-0199-42, lot 4329888) was performed for 30 min at + 4 °C in a 100-μl volume. The cells were then stained with Fixable Viability Dye eFluor 506 (eBioscience, 1:1000, 100 μl/sample) for 30 min at + 4 °C for selecting viable cells. The subsequent steps, including fixation and permeabilization, target probe hybridization with RNA-specific probes, and signal amplification using bDNA constructs, were done as instructed in the PrimeFlow RNA Assay protocol v. 12 July 2017 (Invitrogen, Thermo Fisher). Cells were washed with flow cytometry staining buffer in between stainings and centrifuged 550×*g* 5 min at + 4 °C. Fluorescence minus one samples (FMOs) were included for all fluorophores. Stained single cells were detected using Amnis FlowSight flow cytometry and visualized using IDEAS v. 6.2 software (Merck, Darmstadt, Germany). For all the samples, single round live cells were gated before the analysis based on (1) brightfield channel 1 and IDEAS aspect ratio M01 vs. area M01, (2) area M01 vs. brightfield channel 9 area M09 (to remove additional doublets), and (3) intensity of viability dye below threshold based on an FMO control. Positive signal for all probes was deduced using FMO signals as thresholds. RNA flow analysis was performed with Amnis® FlowSight® imaging flow cytometer (Luminex Corporation, TX, USA).

For the analysis of immunophenotypes in the E/R leukemic bone marrows, archived collected flow cytometry data .fcs files of diagnostic bone marrow biopsies were used. Flow cytometry was performed using Beckman Coulter Navios ten color cytometer. Instrument settings and staining process were done according to EuroFlow SOP [68]. By using the Infinicyt software (Cytognos S.L.), leukemic blasts were gated according to their light scattering characteristics and immunophenotype using two different antibody panels. The first panel included antibodies for TdT, MPO, cyCD3, CD33, CD19, cyCD79a, CD34, CD117, CD7, and CD45, and the second panel for CD66c, CD58, CD10, CD22, CD19, CD123, CD34, CD38, CD20, and CD45. The fluorescence intensities for the antibodies in single events were tabulated and visualized using R/ggplot2. In addition, clinical reports were examined for expert comments on the positivity of each marker (+/−/heterogenous).

For the analysis of NK cell percentages in the diseased bone marrows, flow cytometry was performed during routine diagnostic procedure as above and lymphoid cells were gated and analyzed. The proportions of normal B cells (CD19+, cyCD3−, CD7−), T cells (CD19−, cyCD3+, CD7+), and NK cells (CD19−, cyCD3−, CD7+) from the total normal lymphoid population (utilizing CD45, CD34, and TdT expressions to differentiate from leukemic blasts) were determined from the six E/R-positive patients and from six patients representing other pre-B ALL subtypes (TCF3-PBX1 *n* = 1, high hyperdiploid *n* = 2, B-other *n* = 3 that by transcriptome clustered to BCR-ABL1-like, and DUX4/ERG subtype). On average, 98,564 (min 96,714–max 101,419) live cells were studied in total, including on average 4391 normal lymphocytes in the E/R group samples (1323–10,251) and 5061 (1247–17,101) in the other group.

### Cell proliferation and viability

Effect of drugs targeting TF activities that were found to be high in E/R+ leukemia was studied in the glucocorticoid-resistant REH cell line. The experiments were performed in three biological replicates. TK216 (ERG/FLI1 inhibitor) was acquired from MedChemExpress and XRP44X (Ras-Net-Elk-3 inhibitor) from Sigma-Aldrich. The drugs were reconstituted in DMSO. MTS assay was used to determine viable cells in proliferation upon drug treatments with increasing concentrations at 72 h time point. REH cells (10,000 cells/well) were seeded with drugs into 96-well plates with a final volume of 100 μl. Following drug treatment, cell proliferation was measured using CellTiter 96® AQ_ueous_ One Solution (Promega). Twenty microliters of CellTiter 96® AQ_ueous_ One Solution reagent per well was added, and cells were incubated for 3 h in a humidified (atmosphere 95% air/5% CO_2_) incubator at 37 °C. Absorbance was measured at 492 nm by a spectrophotometer (Thermo Scientific, Multiskan Ex). The background signal (no cells) was subtracted, and the average signal from three technical replicate wells was used in calculations. In parallel, cell viability and count were measured based on Trypan blue (Sigma-Aldrich) staining using Cellometer Mini Automated Cell Counter (Nexcelom Bioscience). Relative proliferation and cell amounts were calculated by normalizing to DMSO as a control sample.

### Visualization tools

Scatter plots and gene set score heatmaps were generated with Scanpy [29] and scVelo [52]. Regulon activity heatmaps were generated with ComplexHeatmap [69]. Illustrations were created with BioRender [70]. Motif logos were generated with HOMER [58]. Track plot from gene loci was generated from UCSC Genome Browser [51] and IGV [71]. Other plots were generated using ggplot2 [72] and base R graphics [73].

## Results

### Bone marrow B-lineage differentiation states are captured in single cell transcriptomes

For a refined view on early B cell differentiation, we processed bone marrow (BM) scRNA-seq data available from HCA [74] and projected each cell into a two-dimensional map using UMAP (see Additional file 1, Fig. S1). A branching map centered at *CD34*+ HSC was obtained, where cycling progenitor cell states led to more differentiated cells that predominantly existed in the G1 cell cycle state based on the cell cycle marker gene scoring (see Additional file 1, Fig. S1a-b), while stromal cells or mature T (*CD3D+*), NK (*GNLY+*), and plasma B cells, which mature outside the BM, clustered separately (see Additional file 1, Fig. S1c).

**Fig. 1:**
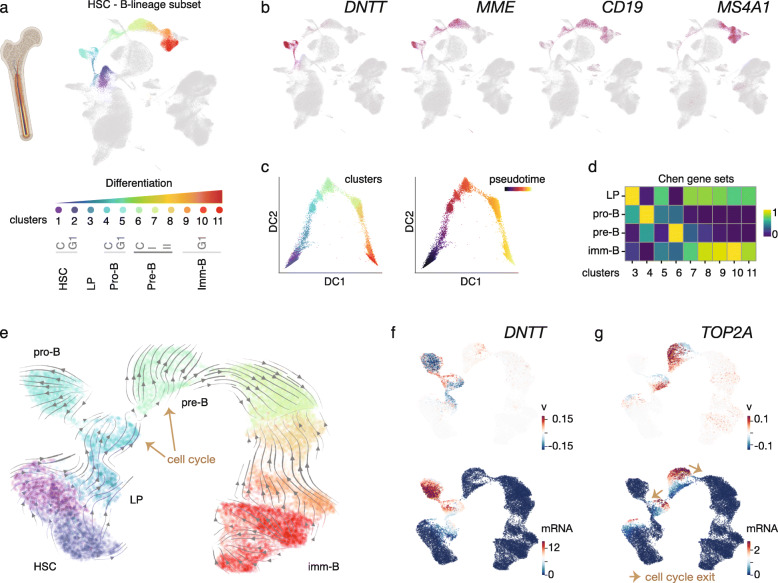
B-lymphoid differentiation states separate in bone marrow scRNA-seq. **a** scRNA-seq clusters for the B-lymphoid lineage defined from HCA BM scRNA-seq data are shown in color on the UMAP visualization and annotated by differentiation and cell cycle stage (C: S/G2/M, G1: G1) (refer to Additional file 1: Fig. S1 for other cell type annotations and cell cycle scoring). LP, lymphoid progenitor. **b** Marker gene expression level is colored on the UMAP, where darker tones of red indicate high expression. **c** Diffusion pseudotime ordering of cells is shown with colors corresponding to clusters shown in **c** (left) or pseudotime (right). DC, diffusion component. **d** Scores for gene sets corresponding to distinct B cell differentiation states [75] are visualized as a heatmap. **e** Differentiation dynamics based on RNA Velocyto analysis is shown for the B-lymphoid cell states. Arrows correspond to predicted direction of cell state transitions. **f**, **g** RNA velocities (top panel) compared to spliced mRNA counts (bottom panel) of the early B cell marker gene *DNTT* and the G2/M-phase specific gene *TOP2A* are shown. Red tones correspond to high velocity or mRNA level, respectively

We separated the B-lineage branch for further analysis, resulting in a reference dataset for B-lineage differentiation from HSCs with 11 clusters (Fig. 1a). The first two clusters corresponded to HSC (in G1 or cycling cell cycle states S/G2/M). *DNA nucleotidylexotransferase* (*DNTT*, also known as TdT) and *MME* (also known as CD10) marker gene expression distinguishes the early lymphoid progenitors (LP, cluster 3) that progress into the *CD19*-expressing cycling and G1 pro-B cell states (Fig. 1b). Furthermore, three pre-B cell clusters (lacking *DNTT* expression) segregated on the map, corresponding to the cycling large pre-B state, followed by pre-B I and pre-B II cells in the G1 cell cycle state (see Additional file 1, Fig. S1d). The pre-B II and the subsequent immature B cell clusters were defined by *MS4A1*(CD20) positivity [76, 77]. The pseudotemporal ordering of the clusters, based on diffusion pseudotime analysis, is shown in Fig. 1c. The progression between cell states based on this analysis is in agreement with the assigned differentiation stages. These cell state annotations had high agreement also with differentiation state scoring using the gene sets defined by flow-sorted B cell populations (Fig. 1d) [75]. However, these gene sets defined from bulk transcriptomes scored highly only in the cycling cell states. Therefore, we additionally distinguished marker genes for each cluster from the single cell analysis (see Additional file 3, Table S2) to facilitate BM B-lineage cell state assignment in future studies. For validation, we processed two independent BM datasets: a healthy adult donor [38] and three pediatric BM samples [36]. From both analyses, we could reproduce the succession of B-lineage clusters observed (see Additional file 1, Fig. S1e).

To delineate the gene expression changes that characterize the cell state transitions in early B-lineage differentiation, we compared the cell clusters sequentially along the pseudotime trajectory (HSC → LP → pro-B → pre-B → immature B cell state). Using the scDD tool [26], changes in mRNA detection (as proportion of zeros), differences in mean expression, and modality could be distinguished for 2201 genes in total with high concordance between HCA and pediatric BM (see Additional file 1: Fig. S2e and Additional file 4: Table S3). Analysis of the RNA dynamics of this gene cohort based on RNA velocity [25, 52] allowed further resolving the B-lineage cell state map (Fig. 1e). In this analysis, both spliced and unspliced counts are used to estimate the velocity of gene expression change, thus extending the cell state representation with gene regulatory dynamics (see the “Methods” section). This is illustrated by *DNTT* (Fig. 1f) that is first upregulated (red tones correspond to positive velocity, top panel) in early lymphoid cells and further increases in mRNA expression (red tones indicating high spliced mRNA counts, bottom panel) at the pro-B state. The pro-B G1 cell state separates as a branch in these analyses, indicating the possibility that this cell state is present as a progenitor pool. Moreover, two successive cycling cell states precede the cell cycle exit into the small pre-B state: the S-phase marker *PCNA* is upregulated (positive velocity) as cells progress from early lymphoid to the first cycling state (pro-B cycling) (see Additional file 1, Fig. S1f) and its mRNA peaks at S-phase cells, coinciding with increasing *TOP2A* velocity (Fig. 1g, top panel, G2/M marker gene) that subsequently peaks in its mRNA level at the G2/M state. The successive increases in the velocity and mRNA levels of these cell cycle state markers indicate the direction of cells on the map and the final exit from the cell cycle into pre-B I G1 state (Fig. 1g, lower panel).

### TF activity changes reveal the regulatory dynamics of B cell differentiation

**Fig. 2:**
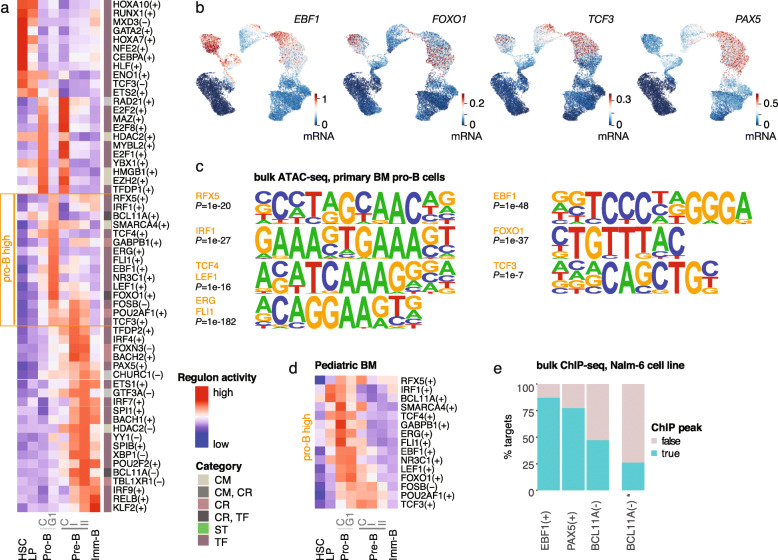
Transcription factor activities across B-lineage differentiation. **a** Regulon activity score is visualized as a heatmap (tones of red indicate high activity). Annotated functional category for regulons: CM, chromatin modifier; CR, coregulator; TF, transcription factor; ST, splicing/transcription complex. +/−: activating/repressive regulatory interaction. Cell cycle stage C = S/G2/M, G1 = G1. **b** Gene expression levels for the TFs *EBF1*, *FOXO1*, *TCF3*, and *PAX5* are indicated in color on the B-lineage scRNA-seq map. **c** Significant motifs matching pro-B active (indicated in **a**) TF regulons are shown from pro-B cell bulk ATAC-seq. **d** Regulon activity score heatmap for pro-B active regulons in pediatric BM. **e** Percentage of TF regulon target genes associated with ChIP-seq peaks is shown for EBF1(+), PAX5(+), and BCL11A(−) regulons obtained with the customized workflow. BCL11A(−)* corresponds to the initial regulon discovered by default SCENIC run

The cell state transitions along the B-lineage trajectory are tightly controlled by TFs. To characterize TF, coregulator (CR), chromatin modifier (CM), and splicing/transcription complex (ST) activities at fine resolution, we performed discovery of the so-called TF regulons with a workflow based on the SCENIC tool [24] (see the “Methods” section for details). Significant predictors for cell states were analyzed by linear model fitting using regulons that were reproducibly identified across training and test set splits. The regulon activity scoring across the B-lineage differentiation stages is shown in Fig. 2a (see also Additional file 5, Table S4) for regulons passing a stringent *R*^2^ cutoff (0.5). Expression levels for TFs involved in the main B-lineage commitment loop (B-lineage TFs reviewed in [78, 79]) are shown for comparison in Fig. 2b. EBF1, FOXO1, LEF1, and TCF4, together with ETS-factors ERG and FLI1, displayed the highest activity (in red) in pro-B cells in our analysis, while TCF3 and PAX5 had similarly high activity in both pro- and pre-B cell states. SPIB and IRF4 activity was elevated later at pre-B cells, together with several negative regulons for TFs with known repressive function such as BCL11A and known co-repressor complex components HDAC2 and TBL1XR1 that interact with glucocorticoid receptor to promote terminal differentiation.

As independent validation, we first retrieved bulk ATAC-seq profiles from pro-B cells [80]. Significantly enriched TF motifs confirmed 9/12 TF regulons (EBF1, FOXO1, TCF3, RFX5, IRF1, TCF4, LEF1, ERG, FLI1) that our analysis associated with the pro-B G1 cell state (Fig. 2c). The pro-B active regulons had a highly similar activity profile also in the pediatric BM dataset (Fig. 2d) (see also Additional file 1, Fig. S3a). Next, we examined closer the regulon gene sets that include TF targets discovered based on TF-to-target gene expression correlation and TF-motif analysis at each target gene locus. We categorized the predicted targets based on how many training/test set splits supported them in the regulon discovery phase. To test whether the predicted targets were bound by the TF, we retrieved ChIP-seq data for PAX5, EBF1, and BCL11A, available in the human cell line model Nalm-6 (see the “Methods” section). Peak to gene associations were obtained using the tool GREAT [66] and compared to SCENIC predictions (see Additional file 5, Table S4). For PAX5 and EBF1, over 75% of predicted targets had a ChIP-seq peak association (Fig. 2e). The validation for the BCL11A repressive regulon was initially low (< 25%). However, upon modification of the regulon discovery strategy (see the “Methods” section; data shown in Fig. 2a corresponds to updated regulon discovery), we could improve this nearly twofold. Moreover, targets discovered across multiple training data splits (Npred, number of iterations supporting the target) were associated with more ChIP-seq peaks (refer to Additional file 1, Fig. S3b), including the most prominent peaks based on ChIP peak score (refer to Additional file 1, Fig. S3c, low ranks correspond to best ChIP scores). The number of associated peaks and their relative peak ranking is further illustrated for top 50 genes from the regulons (refer to Additional file 1, Fig. S3d, targets ranked by Npred). ChIP-seq validated genes include known PAX5 targets from confirmed regulatory loops (*EBF1*, *IRF4*, *BACH2*) and B cell maturation pathways [81–83]. The high agreement of ATAC-seq motif enrichment and the verified TF binding at target gene set loci based on ChIP-seq provides evidence that the TF activity scoring reflects bona fide active regulatory interactions.

In summary, our analysis of healthy BM single cell transcriptomes provides a comprehensive reference for gene expression and TF activity changes that characterize early B-lineage differentiation at single cell resolution.

### E/R leukemic cells resemble the pro-B cell state and display heterogeneity in cell cycle activity

**Fig. 3:**
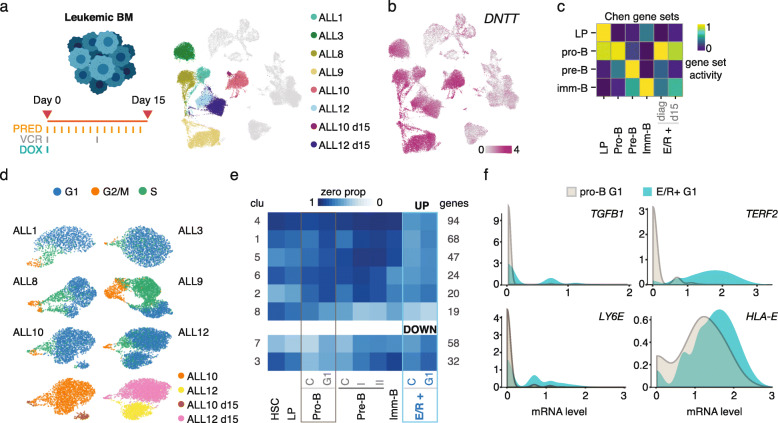
Comparison of E/R+ cells to normal pro-B cells. **a** Six diagnostic and two post-treatment bone marrow samples analyzed with scRNA-seq are shown on the UMAP representation. **b** Expression level of the *DNTT* marker gene is shown in color on the ALL BM UMAP. **c** Gene set scoring of differentiation stage is shown as a heatmap, comparing ALL cells to normal bone marrow lymphoid cells. **d** Computationally predicted cell cycle stage of leukemic cells is colored separately for each donor on a UMAP. For ALL10 and ALL12, the sample origin (diagnostic or day 15 post-treatment) is indicated in the bottom panel. **e** Eight clusters (clu) formed from genes that distinguish ALL cells from normal pro-B cells are shown in the heatmap. The data corresponds to cluster centroids, and the colors indicate the mRNA detection metric ZP (zero proportion), with dark blue tones indicating low expression (high ZP) and light tones corresponding to a larger proportion of cells expressing the genes in each cluster. The number of genes in each cluster is indicated on the right. Cell cycle stage C: S/G2/M, G1: G1. **f** Genes modulating NK cell activity (*TGFB1*, *TERF2*, *LY6E*, and *HLA-E*) plotted as density plots that compare the gene expression distribution of E/R+ cells to pro-B cells (both in G1 cell cycle state)

Lymphoblastic leukemias arise as a consequence of arrested cell differentiation and often carry initiating genetic lesions directly affecting key lymphoid TFs. To characterize leukemic cells carrying the most common TF fusion in ALL (E/R), we performed scRNA-seq on six pediatric E/R+ pre-B-ALL cases, collecting from each the diagnostic BM and from two cases BM at day 15 during induction chemotherapy (Fig. 3a, Table 1). The leukemic cell clusters in each donor were identified based on *DNTT* expression and their clear separation from normal BM cell types (Fig. 3b) (see Additional file 1, Fig. S4a-d and clinical flow cytometry data in Fig. S4e). The normal BM cell populations (erythroid, myeloid, T and NK cells) (see Additional file 1, Fig. S4c) and cycling leukemic states clustered across donors directly, while the similarity of G1 leukemic cells could be ascertained by correcting for donor effect (see Additional file 1, Fig. S4d). Based on the B-lineage cluster-specific gene sets, the diagnostic leukemic blasts resembled the pro-B differentiation state (Fig. 3c). This analysis was supported by label transfer analysis using Seurat [40] (see Additional file 1, Fig. S4c) that similarly identified pro-B cells as the closest normal differentiation state, in agreement with previous studies [4, 7, 84]. The cell cycle state distribution differed between cases, from lowest proportion of cycling cells in ALL3 to highest in ALL9 (Fig. 3d). For the two cases (ALL10 and ALL12) with mid-induction therapy BM profiles, the cells collected at day 15 separated as distinct cell states (Fig. 3a, d), indicating that treatment further alters leukemic cell states.

Next, we aimed to further characterize how the diagnostic E/R leukemic cells differ from pro-B cells by comparing separately the gene expression distributions of cycling and G1 state cells to normal pro-B cells. This analysis was performed against both the HCA healthy BM reference and the pediatric BM pro-B cells. For the majority of genes, the most notable change upon normal B-lineage differentiation was in the zero proportion (ZP) metric that captures the fraction of cells with zero counts for a gene of interest, as exemplified for top 50 genes up- and downregulated in pro-B to pre-B transition (see Additional file 1, Fig. S2e). Therefore, we used ZP for clustering the 272 up- and 90 downregulated genes found in both G1 and cycling cell state comparisons of E/R+ and pro-B cells from HCA (Fig. 3e) (refer to Additional file 4, Table S3 for more extensive gene lists from each comparison). Compared to other cell states along the B-lineage differentiation trajectory, about one third of the upregulated genes were at the highest level in E/R+ cells (cluster 4), while genes in clusters 1, 2, 5, and 6 showed expression in leukemia and normal stem/progenitor cells (Fig. 3e). A smaller fraction (19 genes, cluster 8) were highly expressed in normal pre- or immature B cells, and 16 genes were found significant only in comparison to pediatric BM (see Additional file 4, Table S3). Considering that some gene expression patterns resembled the pre-B cell state, yet the leukemic cells appeared arrested at the pro-B state, we further identified genes that are normally regulated in the pro-B to pre-B transition, to discover additional genes associated with the differentiation arrest. In total, 97 genes normally upregulated upon transition to pre-B state remained at a similarly low level as in normal pro-B cells, while 145 genes downregulated during differentiation remained expressed in leukemic cells (see Additional file 4, Table S3).

Pathway enrichment analysis (see Additional file 6, Table S5) revealed that several of the upregulated genes were associated with cytokine, chemokine, and growth factor pathways, in particular those involved in the negative regulation of NK cell-mediated cytotoxicity. A previous study in ALL implicated elevated TGF-β production in immune evasion [85]. Accordingly, *TGFB1* and three additional genes, *LY6E*, *TERF2*, and *HLA-E*, that contribute to lower NK cell recruitment and activation [86–88] were upregulated in comparison to the expression distribution of E/R+ G1 cells to pro-B G1 cells (Fig. 3f).

### The E/R+ BM immune microenvironment has low abundance and activity of NK cells

**Fig. 4:**
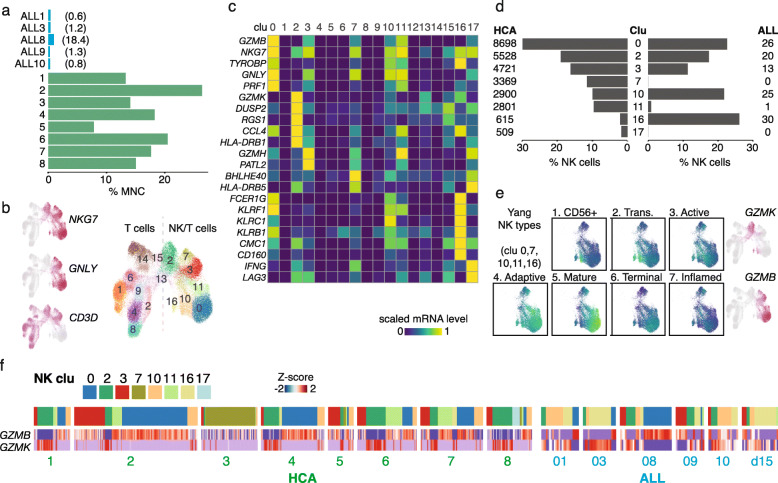
NK cell numbers and activity are low in E/R+ bone marrow. **a** Percentage of mononuclear cells represented by NK cells in BM scRNA-seq data is shown as barplots across the five diagnostic E/R+ ALL (top panel, in blue; ALL12 sample enriched for B cells is not shown) and eight normal BM samples (bottom panel, in green). Percentage of non-leukemic MNCs represented by NK cells is indicated in numbers for the ALL samples. **b** Gene expression level for NK and T cell markers and Louvain clustering of cells is shown on the HCA and ALL NK/T cell UMAP. **c** Marker genes for NK clusters analyzed are shown as a heatmap. Bright yellow color tones correspond to high expression. **d** The percentage of NK cells (in barplot) represented by each cluster from **b** are shown from HCA (left) and ALL (right) samples. Total NK cell numbers assigned to clusters (in HCA or ALL cases) are also indicated. **e** Gene set scores corresponding to scRNA-seq based subtypes [55] are colored on the NK/T cell UMAP. Only cell clusters with low/negative *CD3D* expression used in this comparison are shown. **f** NK cell data plotted separately by donor, indicating the cluster assignment for each cell (colored bar above). The cells (in columns) are clustered based on cluster scores. The heatmap shows the scaled gene expression level for mature vs. immature cell markers *GZMB* and *GZMK*, respectively

The increase in cells expressing genes that may suppress NK cell activity prompted further analysis of the BM immune cells. In accordance, *GNLY* or *NKG7* positive NK cell numbers were markedly reduced in E/R+ BM compared to HCA BM donors (Fig. 4a, percentage of mononuclear cells (MNCs) shown, also seen as reduction in percentage of non-leukemic MNC indicated in numbers). Moreover, according to flow cytometry data, NK cell counts in the lymphoid cell fraction were lowest in the E/R+ vs. non-E/R pre-B-ALL (Welch *t* test *p* value 0.025) (see Additional file 1, Fig. S4f).

To characterize the immune cell populations further, we pooled T and NK cells across the HCA and E/R+ ALL donors for joint analysis. Based on the clustering and marker gene analysis, several different NK cell types could be distinguished (Fig. 4b, c). We focused on clusters expressing *GNLY* or *NKG7* (clusters 0, 2, 3, 7, 10, 11, 16) and noticed that the NK cells from ALL BM were disproportionately assigned to these clusters compared to NK cells from HCA donors (Fig. 4d). Specifically, ALL NK cells mainly represented clusters 10 and 16 that matched granzyme K (*GZMK*) expressing immature CD56^bright^ and transitional NK cells (gene set scores in Fig. 4e represent the NK subtypes from a scRNA-seq study [55]). In comparison, the majority of the normal BM NK cells represented the mature or terminal NK cells (cluster 0) that express granzyme B (*GZMB*) and perforin (*PRF1*). Therefore, E/R+ leukemic cells may actively evade NK cell cytotoxicity. However, the frequency of NK types varied across donors (Fig. 4f). Cluster 7 that expressed *IFNG* at high level corresponded almost exclusively to HCA donor 3, while the highly cell cycle active ALL8 and ALL9 resembled more the mature or active NK profile in normal BM compared to other ALL cases.

Taken together, the leukemic cell states differed from the normal pro-B differentiation state by high expression of stem/progenitor cell-specific genes and several immunomodulatory genes. The changes in immunomodulatory genes were reflected as more immature NK cell types within the E/R+ BM.

### The leukemic regulatory program reveals cell state infidelity in TF activities and includes leukemia risk genes

**Fig. 5:**
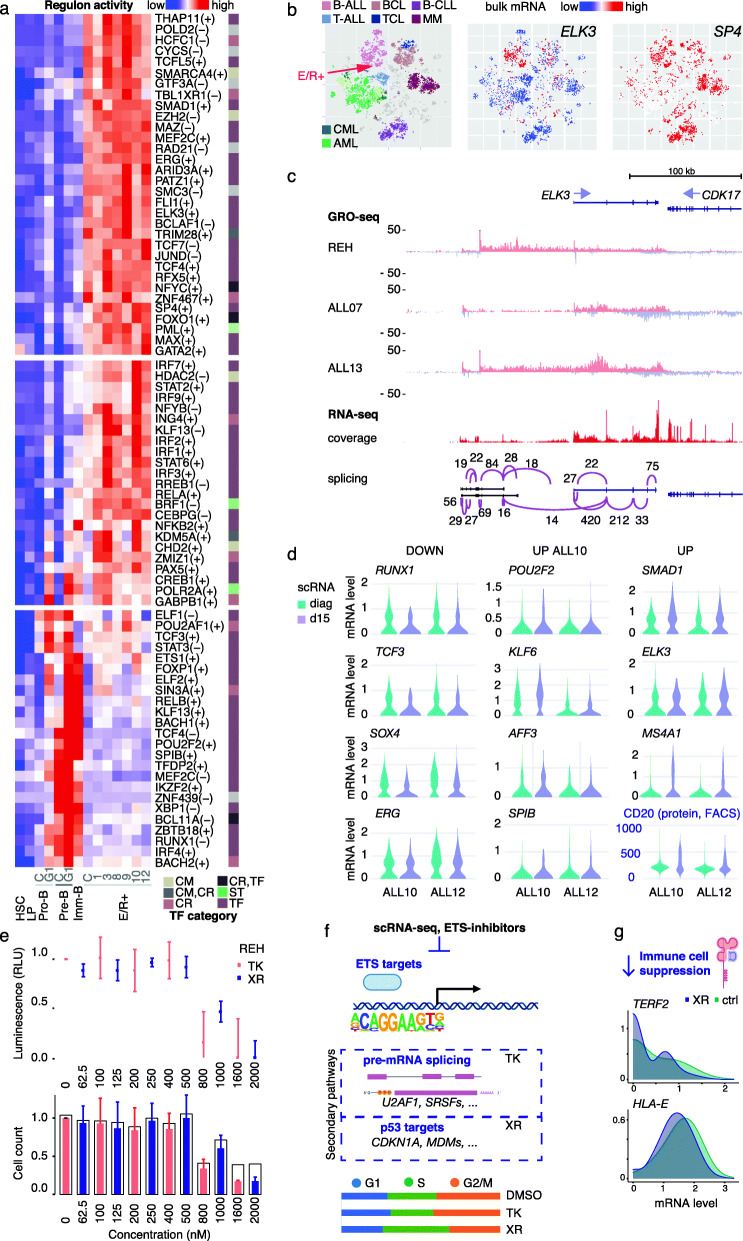
TF activity in E/R+ leukemic cells. **a** Regulon activity is visualized as a heatmap as in Fig. 2a comparing E/R+ cells and normal BM cells. Cell cycle stage C: S/G2/M, G1: G1. Annotated functional category: CM, chromatin modifier; CR, coregulator; TF, transcription factor; ST, splicing/transcription complex. **b** Bulk mRNA expression data for *ELK3* and *SP4* from Hemap is shown on a t-SNE map comparing transcriptomes across hematologic malignancies (ALL, acute lymphoblastic leukemia; BCL/TCL, B/T cell lymphoma; CLL, chronic lymphocytic leukemia; MM, multiple myeloma; CML/AML, chronic/acute myeloid leukemia). The location of pre-B-ALL and E/R+ samples is indicated on the plot. Red color tones indicate high expression. **c** Bulk GRO- and RNA-seq data is shown at the *ELK3* locus. GRO-seq tracks correspond to E/R+ REH cell line and two primary E/R+ bone marrows. Representative RNA-seq signal (coverage) and splicing pattern (Sashimi plot, +strand splicing corresponding to min 10 junction spanning reads) are shown from ALL10. **d** Distributions of expression level at diagnosis and day 15 post-treatment are shown as violin plots for a set of TFs with significant expression change (refer to Additional file 4: Table S3). *Y*-axis corresponds to normalized expression level. The differentiation marker *MS4A1* (mRNA) and CD20 (corresponding protein) level is shown for comparison. **e** The luminescence signal from MTS assay (above) and relative cell counts (viable cells in colored bars, total cell count indicated without fill) at different drug concentrations are shown. TK, TK216; XR, XRP44X). **f** Schematic summary of repressed pathway gene expression upon ETS-inhibition in REH cells (refer to Additional file 4: Table S3). **g** Distributions of mRNA expression level of *TERF2* and *HLA-E* comparing XRP44X treatment to control are shown based on scRNA-seq data in REH

To further decipher the aberrant TF activities contributing to the epigenetic reprogramming that distinguishes E/R+ leukemic cells from normal lymphoid cell states, we repeated the TF regulon activity analysis including the diagnostic leukemic cell states from patient BM (Fig. 5a) (for full list of regulons, refer to Additional file 5, Table S4). Two thirds of the regulons passing the linear model fit (*R*^2^ > 0.5) were active in pro-B cells and showed elevated activity in E/R+ cells, including several ETS-factors (ELK3, ERG, FLI1), FOXO1, MAX, MAZ, SP4, TCF4, and THAP11. However, our analysis also revealed high activity of RFX5 and NFYC in E/R+ blasts that typically would peak only at the immature B cell state. This infidelity in differentiation-stage timed TF activities was also manifested in the misexpression of *GATA2* that is normally confined to HSC and erythroid progenitors. Furthermore, high but more variable levels of IRF, KLF, STAT, and CREB TF family activities characterized the E/R+ cells. Regulons showing diminished activity included RUNX1, SPIB, TCF3, and IRF4 (Fig. 5a).

In further confirmation, we analyzed TF expression matching the positive TF regulons with high activity in E/R+ cells (top panel, Fig. 5a) across large bulk gene expression datasets [67, 89] (Hemap, *N* = 9544, with 1304 pre-B-ALL samples; Pan-ALL, with 1988 pre-B-ALL samples; see also Additional file 1, Fig. S5 and RNA-seq PanALL data in Additional file 5, Table S4). Overall, we could confirm the expression in E/R+ leukemias (log2 signal above probe detection level of approximately 6 (see Additional file 1, Fig. S6)) for all 19 TFs analyzed and 11/19 had significantly higher expression in the E/R subtype.

The ETS-factor *ELK3* and *SP4* have been implicated by genome-wide association studies (GWAS) as risk loci for pediatric pre-B-ALL [90, 91]. Based on the bulk transcriptomes, we could validate their expression in B-ALL, with the highest proportion detected in the E/R+ subtype (red arrow), as shown comparing hematologic malignancies on the t-SNE plot of Hemap samples (Fig. 5b), where lymphoid malignancies are highlighted above the panel (for comparison across B-ALL subtypes, see Additional file 1, Fig. S5). The two most common B-ALL subtypes (E/R+ and high hyperdiploid cases) displayed similarly high *ELK3* while elevated *SP4* was more E/R-specific (Additional file 1, Fig. S5). This prompted further analysis of these TF loci in E/R+ cells (*ELK3* in Fig. 5c, *SP4* in Fig. S6a). Towards this end, we characterized nascent transcription in *E/R+* BM using GRO-seq that reveals engagement of Pol2 to active initiation and elongation at coding and non-coding regions. The GRO-seq profiles confirmed the transcriptional activity of these gene loci in E/R+ cells (Fig. 5c, E/R+ cell line REH and two primary E/R+ bone marrow profiles are shown) (see also Additional file 1, Fig. S6a). Furthermore, it revealed an unannotated (Refseq, UCSC, or Gencode) transcription start site (TSS) upstream the *ELK3* TSS (Fig. 5c). Two lncRNA repositories [92, 93] had matching transcripts within this genomic region; however, the concordance between the annotations was poor (Fig. S6b). Therefore, we further examined the splicing patterns within this locus using paired-end bulk RNA-seq in the E/R+ BM (*n* = 8, matching samples in scRNA-seq and GRO-seq analyses). The annotated *ELK3* transcript had highest support from splice junction spanning reads, while the upstream transcript matched best the MiTranscriptome lncRNA structures (Fig. 5c, data from ALL10 shown). The read-through transcription observed in GRO-seq was reflected in splice events from the upstream transcript to *ELK3*-exon2.

### Leukemic TF activities that persist during chemotherapy provide new targets to overcome resistance

Next, we analyzed the effect of the standard leukemia induction therapy (prednisolone, vincristine, doxorubicin) on the TF expression based on the scRNA-seq profiles acquired at mid-induction therapy in ALL10 and ALL12 (Fig. 5d). Based on differential distribution analysis, residual leukemic blasts from day 15 bone marrow had lower expression of *RUNX1*, *TCF3*, *SOX4*, and *ERG* compared to diagnostic state in both samples, while *SMAD1* and *ELK3* levels increased slightly [refer to Additional file 4, Table S3 for full analysis]. ALL10 had a favorable decrease in blast count at the end of induction on day 29 (0.08%). At day 15, the expression of pre-/immature-B TFs *POU2F2*, *KLF2/6*, *AFF3*, and *SPIB* were elevated in the remaining leukemic cells of ALL10 (10% blasts). These changes may relate to the differentiation-inducing effects of glucocorticoids (daily prednisolone) [94]. However, overall, the changes in TF activities or gene expression were modest, indicating that only partial differentiation towards pre-B cell state may occur, despite the increase in the maturation marker CD20 (encoded by *MS4A1*). In contrast, cases ALL3 and ALL12 responded slowly to therapy (74% and 59% blasts at day 15; 0.16% and 0.2% end of induction, respectively). In ALL3, the cell cycle state distribution was strongly skewed to G0/G1 state at diagnosis (Fig. 3d) compared to the other E/R+ cases, which could underlie resistance to drugs targeting dividing cells (doxorubicin/vincristine). In ALL12, the day 15 sample TF profile indicated persistence of the leukemic gene regulatory program, manifested as a lack of pre-/immature-B TF upregulation (Fig. 5d).

As a strategy to overcome resistance to standard induction therapy, we sought to identify drugs that could target the identified high activity TFs. We selected two compounds for further experiments: XRP44X that has dual activity in targeting microtubules (like vincristine) and simultaneously decreasing ELK3 activation by inhibiting its phosphorylation [95], and TK216 (an analog of YK-4-279 that inhibits ERG and FLI1-mediated transcriptional activity [96]). We used the glucocorticoid-resistant E/R+ REH cells as a cellular model and performed proliferation and viability assays at different drug doses (Fig. 5e) (for dexamethasone control experiment, see Additional file 1, Fig. S6c). At 72 h, cellular ATP levels assessed using MTS assay (top panel) and viable cell counts (bottom panel) dropped sharply at sub-micromolar doses of XRP44X and TK216. Moreover, > 1 μM doses (1.6 μM for XRP44X, 2 μM for TK216) resulted in loss of cellular ATP. To further characterize the drug responses, we selected the drug concentrations 800 nM for TK216 and 1 μM for XRP44X, and prepared scRNA-seq samples. Dead cells were removed during sample preparation. The differential gene expression distributions in surviving cells were compared to control (DMSO-treated) cells separating cells assigned into G1, or cycling phases (see Additional file 4, Table S3). The downregulated genes showed enrichment in ETS-motifs and ETS-factor knockdown signatures, indicating on-target activity of both drugs (see Additional file 6, Table S5). Furthermore, the scRNA-seq data allowed us to characterize secondary pathways that contribute to the drug effect. Overall, the repressed pathways were drug-specific, while many upregulated pathways were shared (see Additional file 1, Fig. S6d). TK216-repressed genes were enriched in functional terms related to splicing and DNA replication (evident also in decrease of S-phase-assigned cells, Fig. 5f bottom panel). In comparison, XRP44X-repressed genes matched p53 target and MHC complex genes, as summarized in Fig. 5f. We further analyzed genes that may contribute to the suppressive BM immune microenvironment (Fig. 3): *HLA-E* and *TERF2* were significantly downregulated upon XRP44X treatment. Both genes were among top predicted targets in ETS-regulons, and we could further support direct binding of ETS TFs to the respective gene regulatory regions based on TF ChIP-seq peak data from several cell types (see Additional file 1, Fig. S6e). In summary, small molecule inhibitors targeting the ETS-factors could be effective in drug-resistant leukemic cells, acting through direct effects on the leukemic regulatory network, cell cycle- and immune-modulatory genes.

## Discussion

Specific cell types are faithfully generated in a repeated manner during development. This is due to gene regulatory interactions that limit the space of stable cell states [97]. Understanding the direct impact of aberrant leukemic TFs on cell state transitions in differentiating lymphoid cells, and identifying TFs that maintain leukemia-specific cell states could enable more precise therapeutic intervention. Here, we explored large-scale single cell transcriptomics data from healthy human BM to generate a reference for cell state transitions and TF activities that characterize early B-lineage differentiation. Focusing on leukemias carrying the E/R fusion, we profiled primary patient BM samples from diagnosis and during induction therapy. The data suggest that the *E/R*+ leukemic cell states resemble most the pro-B state, differ between cases in cell cycle activity, express genes that modulate the immune microenvironment, and may partially be programmed towards pre-B state by induction chemotherapy. Accompanying the differentiation arrest at pro-B cells, our results revealed elevated activity of specific TFs that could serve as therapeutic targets.

Single cell profiling techniques have challenged how we define cell types and provided new methodology to characterize their molecular phenotypes [23, 98]. Previous analysis of the HCA BM data [2, 74] distinguished the B-lineage cell populations but did not further compare them or analyze how the transition from HSC to immature B cells is regulated. One distinguishable feature along this lineage is the alternating cycling and G1 cell populations that the single cell profiling uniquely could resolve. Here, we focused on uncovering key lymphoid TFs orchestrating these cell state transitions. A popular approach to study gene regulation based on scRNA-seq profiles is to analyze the so-called TF regulons defined by TF-to-target correlation and TF motif analysis, available in the SCENIC tool [24]. We benchmarked this method for studying BM cell states, using ATAC-seq motif analysis and target genes for EBF1, PAX5, and BCL11A from ChIP-seq as validation. Compared to the original method, we introduced a cross-validation step and improved capture of repressive TF-target interactions. These regulons faithfully captured targets confirmed by ChIP-seq and TFs that have been previously functionally implicated in B-lineage differentiation through mouse knockout studies [78, 79]. This same analysis strategy could be adopted to identify candidate regulatory programs for cell states across hematologic malignancies.

In this study, we examined the TF activities that may contribute in maintaining leukemic cell states in E/R+ cases and linked those to target genes, including modulators of leukemia-immune cell cross-talk. Previous bulk cancer genomics studies have established that repeated gene expression patterns also characterize cancer samples [99], including ALL where such studies have established several transcriptome-based subtypes [67, 100–103]. They have also shed light on pathway activity and TF expression in E/R+ cells that could be utilized to design targeted therapies [6, 104, 105]. However, the rarity of normal B-lymphoid pro-B cells in BM tissue has represented a challenge to perform direct comparison of E/R+ and healthy BM lymphoid cell states in vivo. Moreover, bulk profiles have obscured the characteristics of the immune microenvironment. Existing scRNA-seq studies in ALL have so far not focused on the leukemic gene regulatory network [36, 106]. Through computational discovery and analysis of TF regulons from scRNA-seq data, and independent validation with bulk genomics data, we could show that elevated activity of multiple ETS-factors (ELK3, ERG, and FLI1) together with pro-B TFs FOXO1, MEF2C, immature B cell TFs NFYC, RFX5, lineage-atypical *GATA2* expression, and E/R subtype-specific SP4 and TCFL5 activities characterized the E/R+ regulatory network. *TCFL5* has been previously shown to be upregulated in E/R+ pre-B-ALL [107–109], while GATA2 has been reported to contribute to the upregulation of erythroid genes, such as *EPOR*, a known marker gene in E/R+ leukemia [110–112]. While these TF activities were consistently high across the six diagnostic samples studied, many IRF- and STAT-regulons showed variable activity. Previously, inhibition of STAT3 was tested in E/R+ leukemic cells and shown to be necessary for *MYC* expression [104]. However, we did not observe correlation between STAT3 and MYC regulon activities in our analysis.

Among the E/R+ TF network, *ELK3* and *SP4* have been reported to confer risk of leukemia development in GWAS [90, 91]. Previous expression quantitative trait loci data from mature B-lymphoid cells indicated that the *ELK3* risk variant associates with its lower expression [90]. This contrasts the data obtained here where high expression was seen in E/R+ scRNA-seq data, which we confirmed by bulk gene expression data comparing across hematologic malignancies [89] and GRO- and RNA-seq profiles in the E/R+ samples analyzed. Comparison across ALL subtypes indicated similar expression levels also in high hyperdiploid pre-B-ALL samples that represent the most common ALL subtype. In E/R+ cells, we observed an active unannotated TSS upstream the *ELK3* locus. By integrating GRO-seq and lncRNA annotations and analyzing splice junctions from RNA-seq, we could match this transcript to a putative exon structure. Furthermore, the genomics data indicated potential read-through and cross-splicing events to *ELK3* exon 2 (harboring the CDS start). Further functional studies on the lncRNA, *ELK3* spliceforms, and the impact of the GWAS risk variants on expression of *ELK3* in normal pro-B cells and leukemia are thus warranted to characterize their role during leukemogenesis. One aspect to study in this context is the role of immune surveillance of pre-leukemic clones, as the target genes that were reproducibly associated with the ELK3 regulon across SCENIC runs included *TERF2* and *HLA-E* that we showed to be highly expressed in E/R+ cells. In addition to HLA-E, class I MHC molecules *HLA-A*, *HLA-B*, *HLA-C*, and *HLA-F* were also upregulated in leukemic cells. Functionally, their expression might interfere with NK cell-mediated tumor surveillance [84, 86–88, 108, 113, 114]. It is known that infection exposure is a key underlying factor in the development of E/R+ leukemias [115–118], thus highlighting the relevance to study the BM immune microenvironment. The decrease in relative NK cell number observed in the E/R+ BM characterized here with scRNA-seq and parallel flow cytometry is in agreement with a larger flow cytometry-based study [85]. However, using scRNA-seq data from E/R+ and normal BM, we could analyze the small NK cell population further. There was a shift towards immature NK cell populations in leukemic BM, and we did not detect subpopulations with high *BHLHE40* or *IFNG* (IFNγ) expression that would characterize active tumor killing, matching targets inhibited by TGF-β [119, 120]. Interestingly, the TF regulons did not indicate canonical activation of SMAD2/3 by TGF-β in the E/R leukemic cells, but instead, both the regulon and differential expression analysis showed high *SMAD1* levels. Atypical activation of SMAD1 via TGF-β has been reported to occur in different cell types [121, 122], and instead of suppressive signaling, it may give E/R+ pre-leukemic cells a growth advantage over healthy pro-B cells [115]. Further changes in innate immune cells (monocytes) were recently reported in an ALL scRNA-seq study [123]. Overall, single cell analyses provide a rationale for carrying out further studies focused on immune cell-leukemia cross-talk to develop therapies that specifically target these immune cell suppressive mechanisms (NK and monocytes) and the detailed genomic characterization of patient material can help to unravel how genetic variations in the leukemia-associated TF loci relate to leukemia risk.

Measurable residual diseases (MRD) at mid [124] and end of induction chemotherapy are predictive markers for relapse risk [13]. Moreover, in vitro resistance to prednisolone has been shown to confer poor prognosis [125]. Previous bulk gene expression studies have indicated treatment-specific changes in gene expression and expression of more mature cell markers [126, 127]. In this study, we sought to gain insight on the efficacy of drug therapy in leukemic cell clearance examining cell state features from scRNA-seq samples collected during in vivo chemotherapy. The E/R+ patient cohort included several cases with residual leukemia cells at mid (day 15) or end of induction (day 29), and we profiled BM samples from two of these at day 15. ALL10 with a favorable end of induction blast count (< 0.1%) regained expression of multiple pre-B/immature B-specific TFs, including *SPIB* and *AFF3*. In contrast, similar changes in TF expression were lacking in blasts (representing 59% of BM cells) in ALL12 at day 15. In ALL3 that also had a high blast count at day 15, the leukemic blasts at diagnosis represented predominantly non-cycling cells. Characterization of these features across a larger patient cohort is thus warranted. To overcome resistance to standard induction therapy, our analysis highlighted candidate drug therapy targets in E/R+ cells that could disrupt leukemic TF activities. Inhibitors abrogating FLI1, MEF2C, ELK3, or SP4 activation have been previously shown to have efficacy in different cancers [95, 128–133]. We tested small molecule drugs targeting the ETS-factors ELK3, or ERG and FLI1 in dexamethasone-resistant E/R+ REH cells and found reduced cell viability with sub-micromolar concentration. We further characterized the drug on-target and secondary pathway activation through scRNA-seq. Both drugs modulated ETS target gene expression, and additionally, TK216 had a repressive effect on splicing-related genes, while XRP44X repressed p53 targets. RNA helicase inhibition by TK216 [133] could underlie splicing changes based on yeast studies reviewed in [134]. Downregulation of p53 targets upon XRP44X treatment, on the other hand, could result from decreased microtubule-mediated p53 nuclear transport [135]. Understanding of these genome-wide drug effects is important for the design and optimum use of cancer therapeutics. As one limitation, our study did not compare the response to other cell types. However, the small molecule ERG/FLI1 inhibitor TK216 tested here has entered a phase 1 study in Ewing sarcoma [136, 137]. Thus, the safety profile from the clinical study could guide further ex vivo and in vivo analysis of this drug in pre-B-ALL. The ELK3 inhibitor (XRP44X) tested has been studied using a mouse model [131] where only limited toxicity was detected.

In this study, we compared the E/R+ leukemic cells to early B-lineage differentiation in healthy adult and non-leukemic pediatric BM. In our analysis, a putative steady state of pro-B cells in G1 state was connected to the succession of cell states from early lymphoid to pre-B state. Pro-B cells can migrate during early development from fetal liver and contribute as a progenitor pool to lymphoid cell generation alongside HSC during early life [80]. As pre-leukemic clones may arise already in utero, the origin and the relative contributions of both HSC- and pro-B pool-derived lymphoid cells at different ages would be relevant to characterize further, which could be achieved using new lineage tracing approaches coupled with scRNA-seq [138–140]. Moreover, compared to other hematopoietic lineages, the succession of lymphoid cell states from early lymphoid to immature B cells differed markedly in transcriptional activity and cell size. The sequential transitions between G1 and cycling cell states pose challenges in single cell analysis in data normalization and resolving the B-lineage differentiation path. Existing benchmarks with downsampling of counts [41, 48] show that normalization methods are robust to differences up to 20% in “size,” yet the differences between G1 and G2/M states observed in lymphoid cell data exceeded this. Moreover, many common trajectory analysis methods fit tree-like structures to data [141]. This challenge motivated our choice of diffusion pseudotime and RNA velocity analyses that both can accommodate cycling cell states [25, 50, 52]. The variability between donors in relative proportions of cycling cells at each differentiation state would also represent a confounder in comparative analysis of cells categorized using differentiation markers alone, as carried out in previous flow-sorted bulk transcriptomes. Therefore, the comparisons of subsequent differentiation states matched by cell cycle state, as performed here, represent a significant advance. One technical confounder in scRNA-seq performed using viably frozen (unfixed) BM samples could derive from the specific protocol used for thawing cells, which could introduce differences in cell populations measured. Using parallel flow cytometry data from thawn cells, we could confirm that different processing steps during library preparation did not alter the leukemic cell content; however, a decrease during freezing occurred in some samples. Therefore, parallel clinical flow cytometry data is valuable and we used it here to confirm changes in the leukemia immune microenvironment. Sample processing could introduce differences also in the transcriptional activity level of cells measured. We noted that the largest variance (PC1) within individual leukemic bone marrow samples reflected their transcriptional activity. These effects could be mitigated by careful selection of analysis steps and underline the importance of good benchmarking data for optimizing single cell workflows for clinical samples.

## Conclusions

This study provides the first comprehensive characterization of cell states and TF activities in E/R+ ALL cases and its comparison to normal human B-lineage differentiation at single cell resolution. We further demonstrate the feasibility of monitoring the early treatment response using single cell genomics and its potential to uncover new therapeutic targets. Through joint analysis of single cell and bulk genomics data, we characterized TF activities contributing to the aberrant cell phenotype in leukemic cells. These results could provide a rational basis for developing new therapies targeting leukemia-immune cell cross-talk and treatment-resistant leukemic cell states.

## Supplementary Information

**Additional file 1: Supplementary figures**. Contains supplementary figures S1-S6 and their legends.

**Additional file 2: Table S1**. Data used in the analysis. Contains data sets used in the analyses and URLs to their repositories and additional metadata and quality controls (flow cytometric analyses, Cellranger quality control summaries).

**Additional file 3: Table S2.** Supporting information related to clustering of cells. Marker genes for clusters in the analysis of B-lymphoid differentiation are listed with statistical analysis of cluster-specificity.

**Additional file 4: Table S3.** Supporting information related to scDD analysis. Cluster membership is indicated for significant genes from leukemic vs. pro-B comparison (against adult and pediatric BM cells), and from drug vs. ctrl (DMSO) cells in the ETS-inhibition experiments carried out in *E/R*+ REH cells. Includes statistical summaries for cell state comparisons related to Figs. 2 and 5.

**Additional file 5: Table S4.** Supporting information related to SCENIC analysis. Summary of regulons discovered in healthy BM B-lineage analysis (related to Fig. 2) and the related validation motif analysis results using pro-B ATAC-seq and regulon target gene analysis for PAX5, EBF1 and BCL11A, including respective ChIP-seq peak data used in validation. Summary of regulons discovered upon including ALL cell states and summary of statistical analysis comparing E/R+ to non-E/R ALL based on bulk RNA-seq (related to Fig. 5).

**Additional file 6: Table S5**. Supporting information related to pathway analysis. Genes from ALL vs. pro-B upregulated clusters or up- and downregulated genes in drug vs. ctrl comparisons (related to Figs. 3 and 5) analyzed using Enrichr. Significant terms summarized from GO, MGI Mammalian Phenotype, BioPlanet Reactome and TF perturbations have a combined score > 150. TF motifs from Genome Browser PWMs are listed with nominal *p*-value <0.05.

## Data Availability

The datasets generated and analyzed in the current study are available in Gene Expression Omnibus under the accession number GSE148218 https://www.ncbi.nlm.nih.gov/geo/query/acc.cgi?acc=GSE148218 [142] and European Genome-phenome Archive under the accession number EGAS00001004374 https://www.ebi.ac.uk/ega/studies/EGAS00001004374 [143]. The Human Cell Atlas bone marrow scRNA-seq data was downloaded from https://data.humancellatlas.org/explore/projects/cc95ff89-2e68-4a08-a234-480eca21ce79 [28]. Pediatric bone marrow scRNA-seq data was downloaded from https://www.ncbi.nlm.nih.gov/geo/query/acc.cgi?acc=GSE132509 [37]. CITE-seq data from an independent adult healthy BM was downloaded from https://www.ncbi.nlm.nih.gov/geo/query/acc.cgi?acc=GSE139369 [39]. Bulk ATAC-seq profiles of pro-B cells were acquired from GEO GSE122989 https://www.ncbi.nlm.nih.gov/geo/query/acc.cgi?acc=GSE122989 [56]. Data for GRO-seq in the REH cell line is available in GEO GSE67540 https://www.ncbi.nlm.nih.gov/geo/query/acc.cgi?acc=GSE67540 [59]. Additional data for ChIP-seq peak analysis was downloaded from GEO GSE45144 https://www.ncbi.nlm.nih.gov/geo/query/acc.cgi?acc=GSE45144 [144], GSE99019 https://www.ncbi.nlm.nih.gov/geo/query/acc.cgi?acc=GSE99019 [62], and GSE126300 https://www.ncbi.nlm.nih.gov/geo/query/acc.cgi?acc=GSE126300 [61]. See also Additional file 2, Table S1 for summary of genomics data used in analyses. Code related to analyses is available from GitHub (https://github.com/systemsgenomics/ETV6-RUNX1_scRNAseq_Manuscript_2020_Analysis) [30].
