# Supplementary material for: Single cell characterization of B-lymphoid differentiation and leukemic cell states during chemotherapy in ETV6-RUNX1-positive pediatric leukemia identifies drug-targetable transcription factor activities
Source: Genome Med. 2020 Nov 20;12:99. doi: 10.1186/s13073-020-00799-2 (PMC7679990; doi:10.1186/s13073-020-00799-2)
Supplement: Supplementary file 1 — Additional file 1: Supplementary figures. Contains supplementary figures S1-S6 and their legends. [file 13073_2020_799_MOESM1_ESM.pdf]

## Additional file 1

Single cell characterization of B-lymphoid differentiation and leukemic cell states during chemotherapy identifies drug-targetable transcription factor activities in ETV6-RUNX1 positive pediatric leukemia

## Supplementary Figures

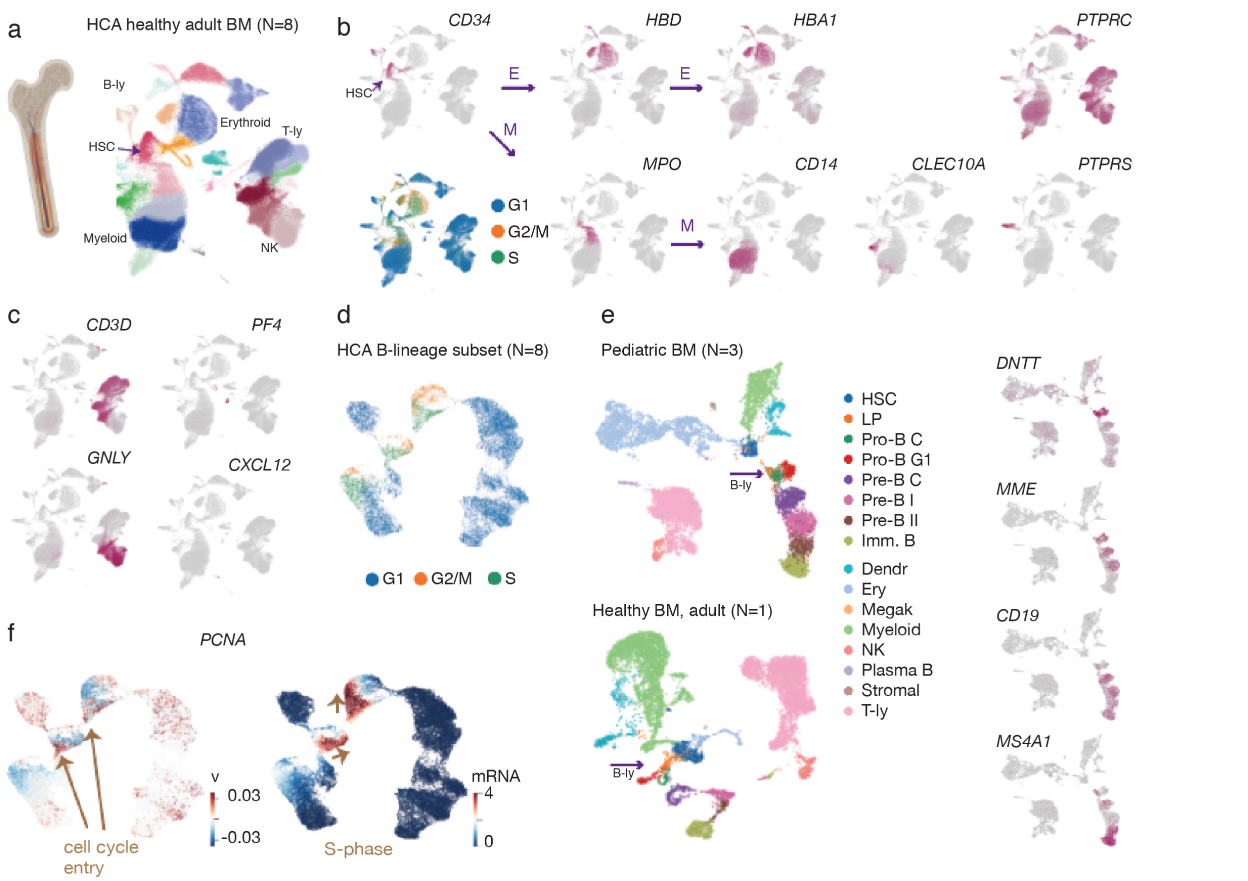


**Figure S1. HCA BM dataset**. a. Initial cell state assignment of Louvain clusters is presented on the two-dimensional UMAP visualization of HCA BM scRNA-seq (N=8). b-c. Expression level for classical lineage marker genes and the computationally predicted cell cycle state are visualized on the UMAP: stem cell (*CD34*), E: erythroid (*HBD*, *HBA1*), M: myeloid (MPO, CD14, CLEC10A for classical and PTPRS for plasmacytoid dendritic cells), T-lymphoid (*CD3D*), NK cell (*GNLY*), megakaryocyte (*PF4*) and *PTPRC* (also known as *CD45*) negative stromal (*CXCL12*) cells. d. Computationally predicted cell cycle state is shown on the HCA B-lineage subset UMAP. e. UMAP visualization of two independent BM datasets where the lineage or the more specific differentiation stage (for B-lineage) label (left) is colored. For pediatric BM also the B-lineage differentiation marker genes shown in Fig. 1b are colored on the map (right). f. RNA velocity (left) and mRNA expression (right) for the S-phase marker *PCNA* are shown as in Fig. 1f.
*Related to Fig 1.*

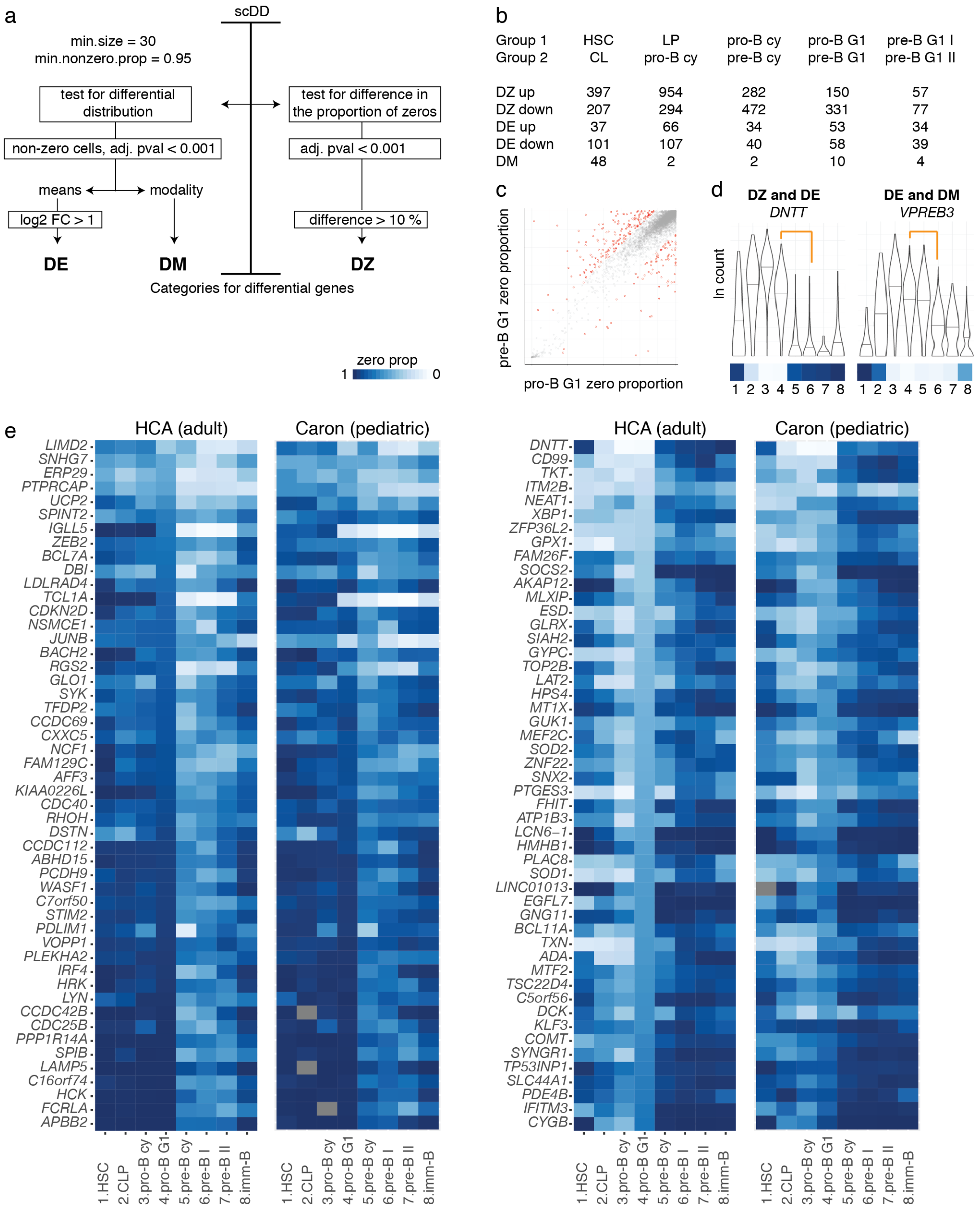


**Figure S2. Differential distribution testing between normal BM cell population using the scDD tool.** a. A schematic representation of filtering steps and cut-offs applied in the scDD analysis. See Methods for details. b. Number of differential genes along B-cell differentiation from HSC to preB-G1 II cell state (HCA) is shown as a table. c. Scatter plot of ZP in pro-B G1 (x-axis) and pre-B G1 (y-axis) cell populations is shown (HCA data). Red color indicates genes assigned to the DZ category. d. Violin plots for example genes showing the gene expression distribution found in the pro-B G1 to pre-B I comparison (refer to panel e numbering) in both DZ and DE analyses but not DM (*DNTT*), and in both DE and DM but not DZ (*VPREB3*). e. Top 50 genes up- and downregulated in pro-B to pre-B transition (in both G1 and cycling cell population comparisons) are shown as a heatmap comparing the adult (HCA) and pediatric (Caron et al) datasets. Color corresponds to ZP and darker tones indicate a smaller portion of cells expressing the gene. *Related to Figs. 1, 3, 5*

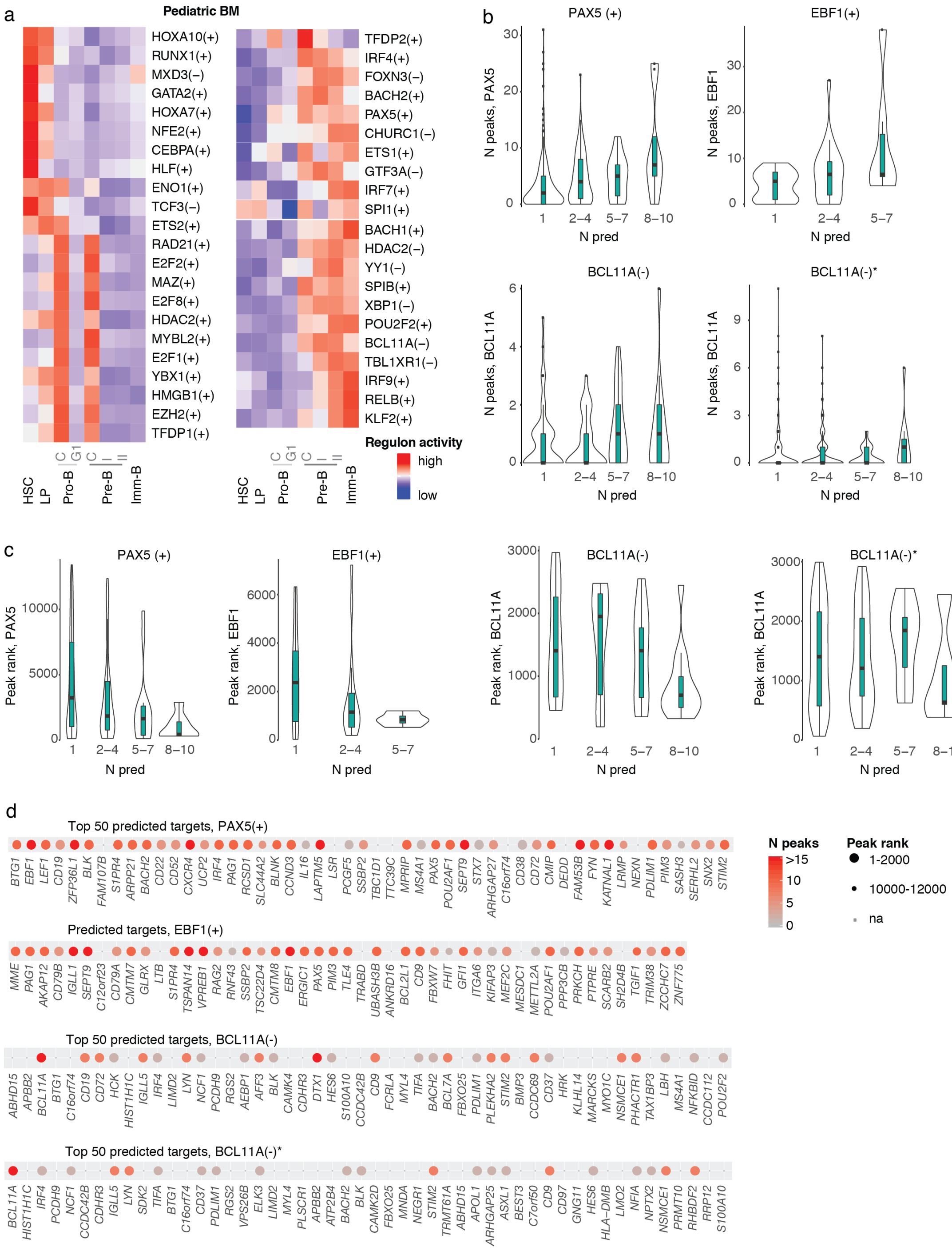


**Figure S3. ChIP-seq validation data for SCENIC regulons.** a. Regulon activity score heatmap for the remaining regulons, as in Fig. 2a, for pediatric BM data. b. The distribution of ChIP-seq peaks associated to targets is shown as a combined violin and box plot for predicted targets of EBF1(+), PAX5(+) and BCL11A(-) regulons obtained with the customized workflow. BCL11A(-)* corresponds to initial regulon discovered by default SCENIC run. c. The ChIP-seq peak rank distribution is visualized across binned regulon genes. Lower rank corresponds to higher ChIP peak score. d. The ChIP peak data is visualized using a dot plot for top 50 predicted targets. The color corresponds to the number of associated peaks and the dot size indicates binned ChIP-peak rank (bin size 2000). *Related to Fig. 2*

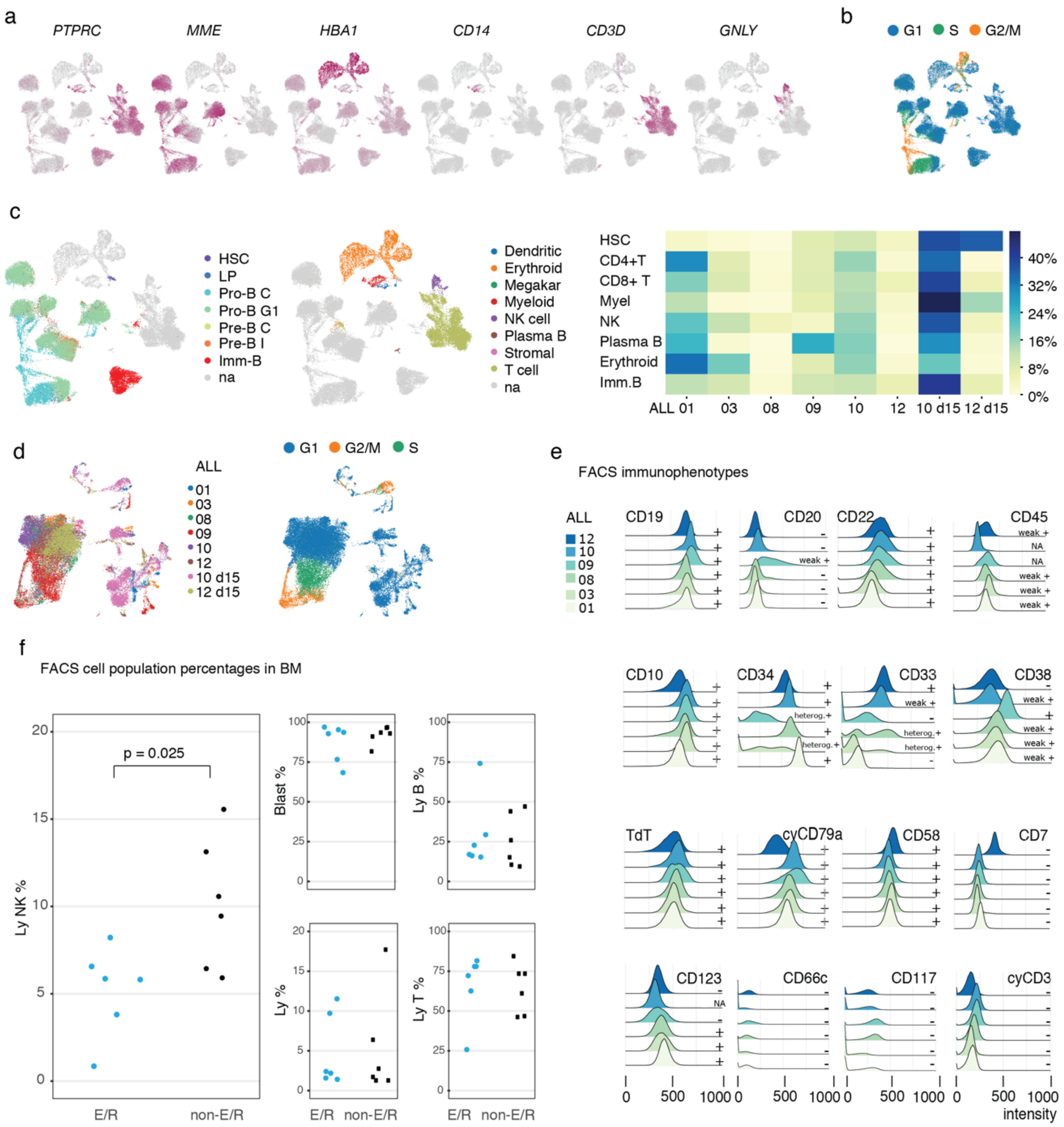

**Figure S4. E/R+ BM dataset.** a. Expression level for classical BM marker genes visualized on the UMAP: mature blood cell (*PTPRC*), B-lymphoid progenitor (*MME*) erythroid (*HBA1*), myeloid (CD14), T-lymphoid (*CD3D*) and NK cell (*GNLY*) cells. b. Computationally predicted cell cycle state presented on the UMAP. c. Cell type assignment is shown for the E/R+ BM UMAP based on label transfer from HCA BM annotations (left: B-lineage cell states, middle: other BM cell types). The contribution of each sample to non-leukemic cell types is shown as heatmap (right). Diagnostic samples contribute to each cluster. The non-depleted post-treatment sample contributes majority of cells. d. Sample origin (upper panel, donor in color) and computationally predicted cell cycle state (lower panel) are shown on the E/R+ BM batch-corrected UMAP (batch=donor). The largest cluster on the left (with cell cycle status G1 above and G2/M/S below) corresponds to leukemic cells. e. Immunophenotypes based on clinical flow cytometry shown as ridge plots. Annotation (+/-/heterogenous) is based on comments in clinical reports. f. Non-leukemic lymphoid fraction cell populations compared between E/R+ and non-E/R ALL BM samples based on clinical flow cytometry. The Welch two sample t-test p-value is indicated for NK cell percentage from lymphoid cells. Ly% = percent of normal lymphoid cells of all alive cells. Ly_B, Ly_T, Ly_NK % = percent of B-, T-, or NK-cells of all normal lymphoid cells. *Related to Fig. 3*

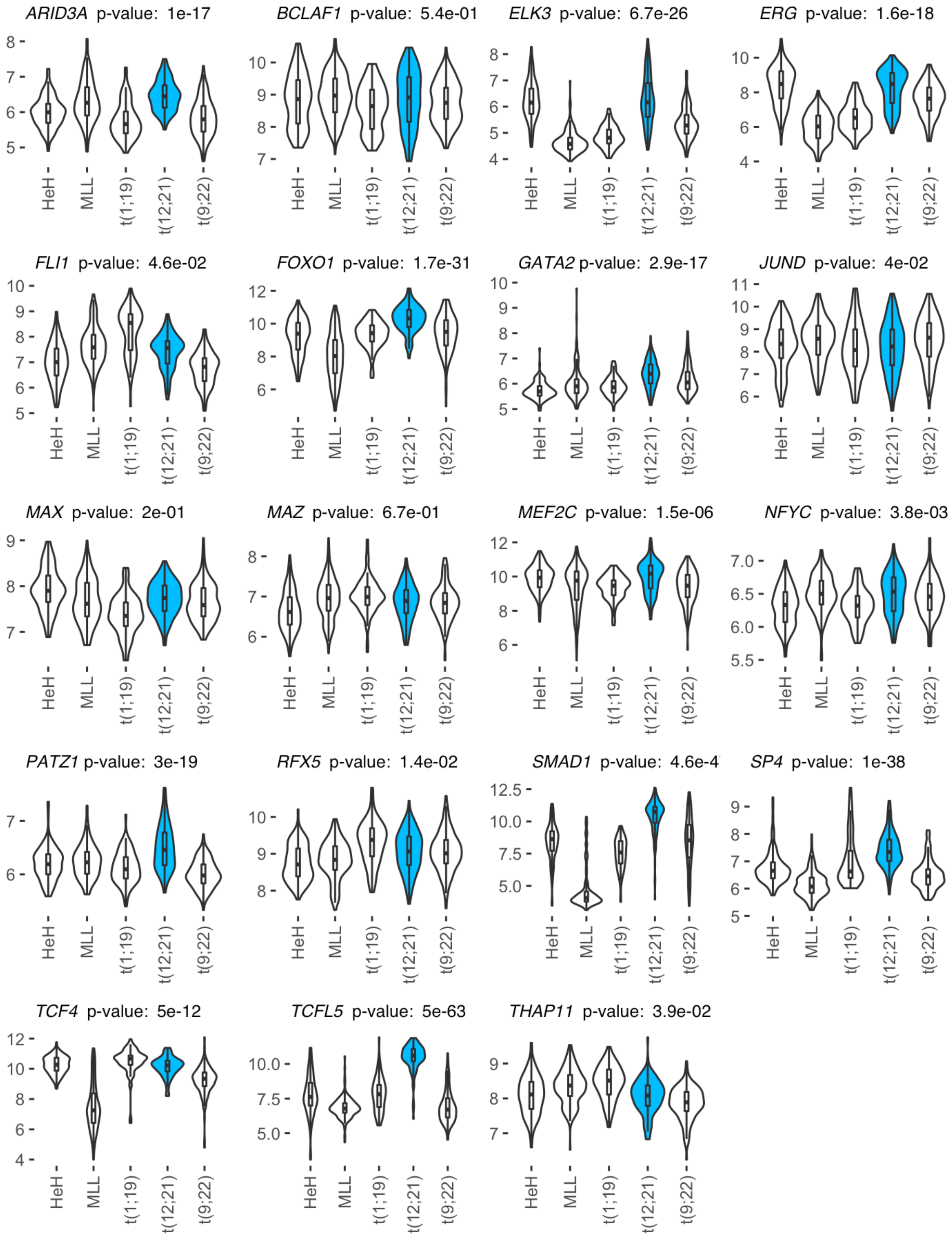


**Figure S5. Bulk gene expression data for pre-B-ALL subtypes**. The log2 gene expression levels in Hemap microarray dataset compared across pre-B-ALL subtypes as boxplots. TFs with high predicted TF activity in E/R+ ALL are shown. Two-sided Welch t-test p-values are indicated for E/R+ vs non-E/R comparison. *Related to Fig. 5*

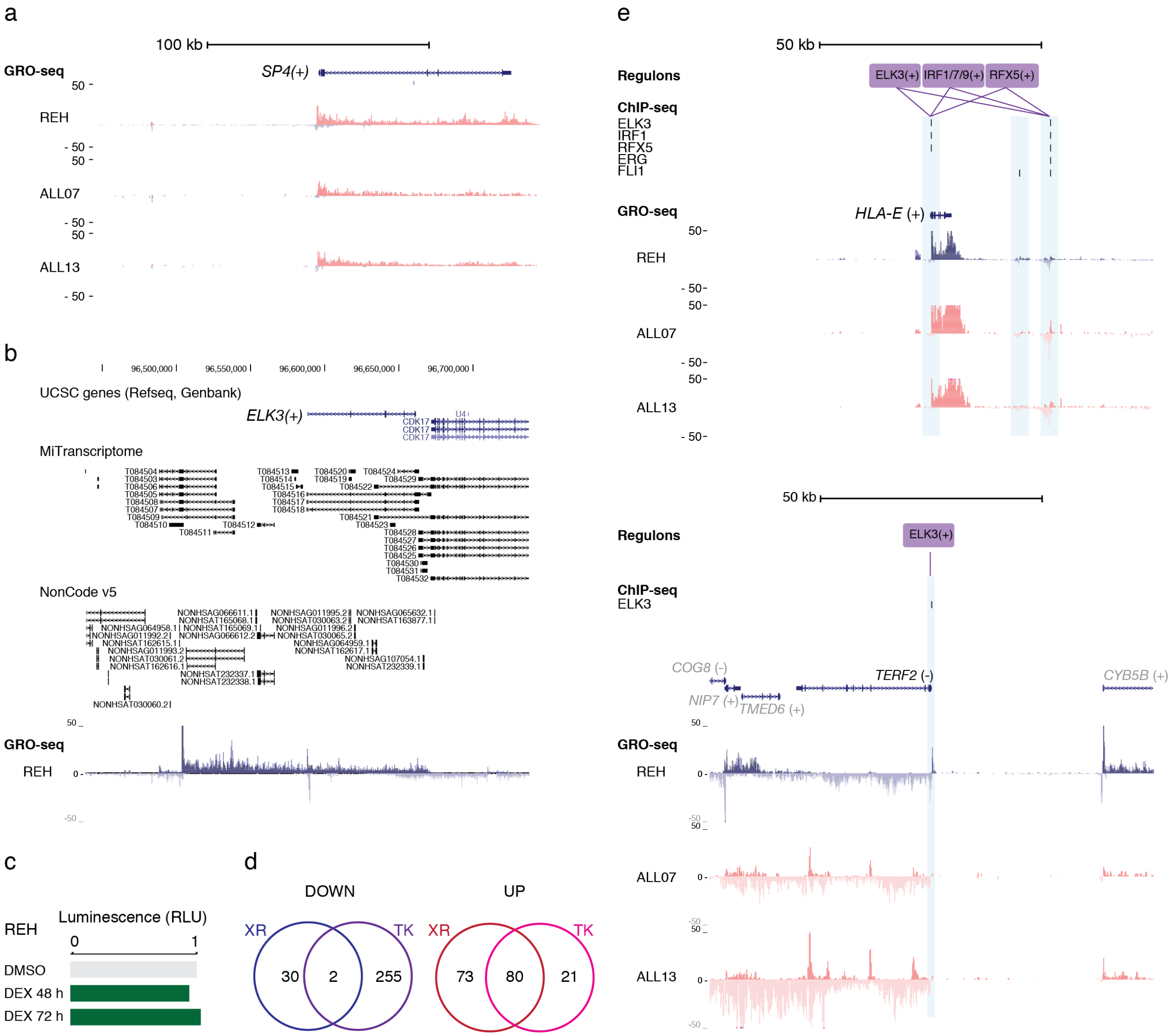


**Figure S6. Characterization of active TF regulon TF and target genes in E/R+ cells**. a. GRO-seq data shown at the *SP4* locus as in Fig. 5c. b. Gene and lncRNA annotations at the *ELK3* locus. c. The luminescence signal from MTS assay in REH cells treated with the glucocorticoid dexamethasone (10 nM) is shown from 48 h and 72 h time points. d. Venn diagrams comparing down- and upregulated pathways (scRNA-seq) between the ETS-inhibitors XRP44X and TK216. e. ChIP-seq and GRO-seq data are shown at the *HLA-E* and *TERF2* loci as in Fig. 5c. Active enhancers are highlighted by shading. The ChIP-seq peaks shown at these locations correspond to HUVEC (ELK3), HSC (ERG, FLI1), K562 (IRF1 upon IFNg stimulus) and GM12878 (RFX5) peak annotations. Lines between enhancer or promoter regions and regulons are drawn when a corresponding peak was found. GRO-seq data is shown from E/R+ REH cell line and two primary E/R+ bone marrows. *Related to Fig. 5*
